# Conformation-Determined Segmental Dynamics in α‑Poly‑l‑Lysine Hydrobromide: The Impact of Thermally Induced β‑Sheet Formation on the Glass Transition Temperature

**DOI:** 10.1021/acs.jpcb.6c03934

**Published:** 2026-08-06

**Authors:** Rafał Bielas, Anjana Krishna Sudhakaran Nair Valsala Kumari, Krzysztof Witkowicz, Anna Mielańczyk, Magdalena Tarnacka, Anna Janowska, Karolina Jurkiewicz, Kamil Kamiński, Barbara Hachuła, Ewa Kamińska

**Affiliations:** † Department of Pharmacognosy and Phytochemistry, Faculty of Pharmaceutical Sciences in Sosnowiec, Medical University of Silesia in Katowice, Jagiellońska 4, 41-200 Sosnowiec, Poland; ‡ A. Chełkowski Institute of Physics, Faculty of Science and Technology, University of Silesia in Katowice, 75 Pułku Piechoty 1, 41-500 Chorzów, Poland; § Institute of Chemistry, Faculty of Science and Technology, University of Silesia in Katowice, Szkolna 9, 40-006 Katowice, Poland; ∥ Department of Chemistry, Silesian University of Technology, B. Krzywoustego 4, 44-100 Gliwice, Poland

## Abstract

Solid-state polypeptides undergo continuous conformational reorganization when subjected to various environmental stimuli, reshaping their macroscopic physical behavior. Here, we investigate this phenomenon specifically under thermal treatment using α-poly-l-lysine hydrobromide (α-PLL), an ionic polypeptide containing Br^–^ counterions, as a model system. By combining thermogravimetric analysis (TGA), Fourier-transform infrared (FT-IR) spectroscopy, wide-angle X-ray scattering (WAXS), differential scanning calorimetry (DSC), and broadband dielectric spectroscopy (BDS), we show that freeze-dried α-PLL initially contains mixed α-helical, β-sheet, and β-turn/antiparallel β-sheet conformations. After removal of loosely bound water, thermal treatment drives a transformation toward β-sheet-rich structures. This process is cooperative and thermally activated, with an apparent activation energy of approximately 64 kJ mol^–1^, indicating that β-sheet formation requires collective rearrangement of hydrogen-bonded polypeptide chains. The structural transformation is not limited to local changes in secondary structure. WAXS reveals that α-PLL exhibits liquid-crystalline-like two-dimensional hexagonal packing, which is reorganized in a molecular-weight-dependent manner as β-sheet-rich architectures develop with increasing temperature. Most importantly, this conformational reorganization produces two unexpected changes in the physical response of α-PLL. First, the formation of β-sheet-rich structures is accompanied by a decrease in glass transition temperature (*T*_g_), showing that secondary-structure ordering does not necessarily rigidify the polypeptide matrix. Second, the frequency-dependent conductivity, σ′(f), systematically decreases over a broad frequency range upon annealing, although the β-sheet-rich state exhibits stronger hydrogen bonds. This suggests that charge transport in α-PLL is promoted not by the stronger, more static interchain hydrogen-bonding network of β-sheets, but by the α-helical state, which may provide a more favorable electrostatic environment for Br^–^ migration. These findings reveal a direct link between secondary-structure transformation, glass-transition dynamics, mesoscale packing, and ion transport, establishing α-PLL as a model solid-state polypeptide electrolyte in which conformational reorganization governs both molecular mobility and electrical response.

## Graphical abstract

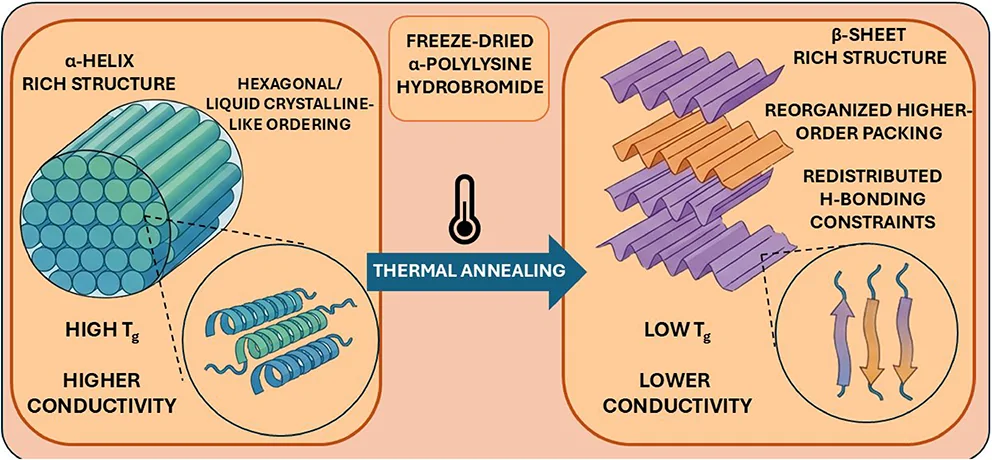

## Introduction

Amino acid–based biopolymers, and particularly synthetic polypeptides, constitute an important class of functional soft materials that bridge the gap between structurally complex natural proteins and chemically tunable synthetic polymers.^1,2^ Their particular value lies in the fact that they preserve the essential conformational motifs of proteins, including α-helices, β-sheets, β-turns, and random coils, while avoiding the complexity associated with tertiary and quaternary folding.^3–5^ Because these secondary structures govern biological activity, physicochemical behavior, and materials performance, simple synthetic polypeptides provide controlled model systems for examining how peptide chains reorganize, aggregate, and respond to external stimuli.^6–8^ This makes them useful not only for the design of responsive peptide-based materials, but also for understanding conformational processes that are difficult to isolate in complex protein architectures. Such conformational rearrangements are particularly relevant to amyloid-β, tau protein, α-synuclein, and prion proteins, for which structural transitions toward β-sheet-rich states are associated with pathological aggregation. These processes are implicated in proteopathic neurodegenerative disorders, including Alzheimer’s disease, Parkinson’s disease, and prion diseases.^9–23^

In this context, α-poly-l-lysine (α-PLL) is a particularly useful model homopolypeptide. It is a linear polymer composed of l-lysine residues connected by peptide bonds,^24^ and it can adopt several chain conformations, most notably α-helical, β-sheet, β-turn, and random-coil-like states. These conformations differ in hydrogen-bonding pattern, intermolecular organization, physical properties, and biological activity. At the same time, α-PLL is biocompatible, biodegradable, and cationic, which has led to its use in nanomedicine and biotechnology, including as a nonviral RNA carrier, an antibacterial agent, a bioactive coating, and a bioink component.^25–33^ Thus, α-PLL combines practical relevance with structural simplicity, making it an attractive platform for studying how secondary structure controls the properties of peptide-based materials. The conformational behavior of α-PLL has been investigated for decades, mainly using circular dichroism (CD) and Fourier-transform infrared spectroscopy (FT-IR). Previous studies have shown that the final structure depends strongly on preparation history. Slow drying generally favors the thermodynamically stable β-sheet arrangement, whereas rapid drying or stabilizing additives can preserve more disordered or random-coil-rich states.^34^

Freeze-drying has been reported to yield mixtures of α-helical and β-sheet conformations, whereas spray-drying may further promote an α-helix-to-β-sheet transformation.^35^ In some reports, this transformation is referred to as denaturation; however, in the case of α-PLL, this term is somewhat misleading, as the process predominantly involves reorganization between different secondary structures, rather than the conventional unfolding of a native globular protein. The broader literature on conformational changes in α-PLL has been dominated by studies in aqueous solution, where pH, hydration, ionic environment, temperature, and shear are known to shift the balance between α-helix, β-sheet, and random coil conformations.

Among these factors, temperature is particularly important because it can directly promote the α-helix-to-β-sheet transition by weakening intrachain H-bonds, increasing chain mobility, and facilitating interchain association. CD studies in alkaline buffer showed a sharp decrease in α-helical content above approximately 318 K, accompanied by a concomitant increase in β-sheet fraction and only minor changes in random-coil content.^36^ This behavior indicates that heating does not simply disorder the polypeptide chain, but rather drives its reorganization toward a more extended and intermolecularly associated β-sheet-rich state. A similar temperature-sensitive phenomenon was observed in a α-PLL in deuterated water, where the α-helix-to-β-sheet transition has already been reported near room temperature.^37^ Other experiments demonstrated that shear can also promote this conformational conversion, further emphasizing that β-sheet formation in α-PLL is governed by a combination of dehydration, side-chain interactions, enhanced chain mobility, and interchain aggregation.^37,38^ Overall, these studies established that α-PLL is highly sensitive to its local environment and that the thermally promoted α-helix-to-β-sheet transition is one of its central conformational pathways.

However, despite this extensive literature, much less is known about conformational transformations of α-PLL in the solid state. This is a significant limitation because the solid state provides access not only to secondary structure, but also to fundamental physical properties that are not affected by additional interactions with the solvent. One of the few solid-state thermal aging studies on α-PLL films showed an α-helix-to-β-sheet transition after annealing at 296–403 K for 30 min, accompanied by a random-coil-to-antiparallel-β-sheet reorganization at higher temperatures.^39^ This result is important because the antiparallel β-sheet has been identified as the energetically favored structure.^35^ Yet, because kinetic measurements were not performed, the time-dependent pathway of this transformation remained unresolved.

Solid-state α-PLL has also attracted attention from the perspective of molecular mobility and hydration. Early studies estimated the glass transition temperature (*T*_g_) of this polymer near 323 K,^40^ while hydrated α-PLL was shown to contain surface-bound water that remains noncrystalline down to 180 K and strongly plasticizes the peptide matrix.^41^ More recently, broadband dielectric spectroscopy (BDS) was used to study Cbz-protected α-PLL, selected for its reduced ionic conductivity.^42^ That system exhibited a well-defined glass transition near 305 K and non-Arrhenius relaxation dynamics. Further pressure-dependent measurements allowed authors to interpret the glass transition as an intrinsic “thawing” of backbone segmental mobility, largely independent of the specific secondary structure or local H-bonding pattern.^42^

As mentioned above, studies on α-PLL as a solid-state polypeptide system are rarely performed—an oversight that is heavily unjustified considering that this polymer is emerging as a cornerstone material for bioelectronics,^43–45^ advanced sensors,^46–49^ and high-stability pharmaceutical matrices.^50,51^ The performance of these applications is dictated by the material’s bulk properties; specifically, the mastery of glass transition and conductivity within the solid matrix is paramount to ensuring functional reliability. However, the lack of systematic research in this area leaves several key questions unresolved: What is the time scale and activation barrier for the conformational change in solid-state PLL? How does thermally induced conformational reorganization affect macromolecular dynamics? In particular, the kinetics of secondary-structure evolution and its impact on the basic properties of solid α-PLL have not been systematically examined. Addressing this gap is essential to understanding whether the glass transition in peptide-based polymers is truly independent of secondary structure or whether it is directly modulated by conformational order and intermolecular H-bonding.

In this work, we present a systematic study of two α-poly-l-lysine hydrobromide samples, denoted **PLL1** and **PLL2**, with different molecular weights, using FT-IR spectroscopy, wide-angle X-ray scattering (WAXS), differential scanning calorimetry (DSC), and BDS. For simplicity, these hydrobromide salts are hereafter referred to as α-PLL, **PLL1**, and **PLL2**. By combining structural, thermal, and dielectric probes, we follow the thermally induced evolution of α-PLL from initially mixed conformational states toward β-sheet-rich architectures and determine how this transformation affects macromolecular mobility. We show that the conformational transformation is irreversible, temperature-dependent, and accompanied by the formation of intermolecular β-sheet structures. Most importantly, our results demonstrate that *T*_g_ and dielectric response are intrinsically coupled to polypeptide conformation, calling for a reconsideration of the previously proposed independence of glass-transition dynamics from secondary structure.^42^ Uncovering this relationship provides a vital blueprint for the design and scalable production of next-generation sensors, bioelectronics, and biopharmaceuticals.

## Materials and Methods

### Materials

*N*-carbobenzoxy-l-lysine (H-Lys­(Cbz)–OH) and triphosgene were purchased from Fluorochem. Propylene oxide was acquired from ThermoFisher. Tetrahydrofuran (THF), *N*,*N*′-dimethylformamide (DMF), and diethyl ether were obtained from Chempur. 2-Ethylhexylamine was purchased from Sigma-Aldrich. Hexane was purchased from POCh, Avantor. Deuterated dimethyl sulfoxide (DMSO) and chloroform (CDCl_3_) were purchased from Sigma-Aldrich.

### Preparation of Lys­(Cbz) NCA

H-Lys­(Cbz)–OH (10 g, 35.67 mmol) and propylene oxide (19 mL, 0.383 mol) were suspended in 150 mL of dry THF and stirred. Next, triphosgene (5.5 g, 18.53 mmol) was added to the suspension. The suspension was stirred at 293 K until all solids disappeared and the solution became clear (approximately 2 h). The solution was then concentrated under reduced pressure, filtered, and then precipitated by the addition of 800 mL of hexane, and stored overnight at 253 K. The *N*-carboxyanhydride (NCA) powder was dried, redissolved in dry ethyl acetate, filtered, and reprecipitated again in hexane. Next, it was vacuum-dried to afford a colorless solid (yield 83%).

### Preparation of α-Poly-l-Lysines (α-PLL) via Ring Opening Polymerization of NCA

H-Lys­(Cbz)–OH *N*-carboxyanhydride (Lys­(Cbz) NCA; 5 g; 16.3 mmol) was placed in a Schlenk reactor equipped with a magnetic stirrer, followed by the addition of 15 mL of DMF and stirring until complete dissolution. 2-Ethylhexylamine (96.4 μL; 76 mg; 0.652 mmol for **PLL1** or 53.5 μL; 42.2 mg; 0.326 mmol for **PLL2**) was then added. The reactor was capped, and the system was placed under vacuum. The reaction was monitored by FT-IR spectroscopy and terminated after the absorption bands corresponding to the anhydride had completely disappeared. The polymer was precipitated in diethyl ether and a small portion of the protected precursor polymer was collected at this stage for SEC analysis. The remaining material was then dissolved in trifluoroacetic acid (20 mL), and a 33% solution of hydrogen bromide in acetic acid (4 mL) was slowly added dropwise. After stirring for 24 h, the polymer was reprecipitated in diethyl ether. The precipitate was then dissolved in deionized water. The solution was placed on a dialysis membrane (MWCO = 3.5 kDa) and dialyzed (7 days, with water changed three times daily or until the acidic pH disappeared). After dialysis, the solution was freeze-dried. A white powder (3.23 g) was obtained. The resulting deprotected polymer was characterized by ^1^H NMR.

### ^1^H Nuclear Magnetic Resonance (NMR)

^1^H NMR spectra were obtained using a Bruker Ascend 600 MHz instrument. Samples were prepared in deuterated DMSO and CDCl_3_.

### Size-Exclusion Chromatography (SEC)

The SEC measurements were performed using an Ultimate 3000 chromatograph (Thermo Fisher Scientific in Waltham, MA, USA), paired with a RefractoMax 521 differential refractometer detector. The Cbz-protected PLL precursor samples, diluted in HPLC-grade DMF containing 10 mM LiBr, were analyzed at 323 K through a TSKgel Guard SuperMPHZ-H 6 μm precolumn (4.6 mm × 2 cm) and two TSKgel SuperMultiporeHZ-H 6 μm columns (4.6 mm × 15 cm) at a flow rate of 0.25 mL/min. Calculations of apparent *M*_n_ and *Đ* were based on PEO standards ranging from 3060 to 504,000 g/mol.

### Fourier-Transform Infrared (FT-IR) Spectroscopy

FT-IR spectra were recorded using a Nicolet iS50 spectrometer (Thermo Fisher Scientific, Massachusetts, USA) equipped with a DLaTGS detector. The measurements were performed at a spectral resolution of 4 cm^–1^, accumulating 16 scans for each spectrum. Initial vibrational analysis of the **α-PLL** samples was conducted in ATR mode within the 4000–400 cm^–1^ wavenumber range at room temperature (293 K).

To investigate the temperature-induced conformational transitions, high-temperature FT-IR data were collected using a GladiATR accessory (Pike Technologies) across the same spectral range. To ensure high spectral accuracy and compensate for thermal effects, background spectra were recorded at each specific temperature and automatically subtracted during sample acquisition. Before the proper experiment, the samples were annealed at 393 K (**PLL1**) and at 353 K (**PLL2**) for 15 min to remove residual water, then cooled back to room temperature. Representative IR spectra of the samples, before and after annealing, are presented in the Supporting Information (SI), Figure S4. Then, the subsequent temperature investigations were performed by heating the samples from 293 to 453 K, with spectra recorded at 4 K intervals. For the kinetic studies, time-dependent IR spectra of **PLL2** were collected under isothermal conditions at selected temperatures (383, 393, 403, 413, 433, and 453 K) as well as at 393 K for **PLL1** to monitor the evolution of the amide I band (1750–1580 cm^–1^) over time. During all measurements, the optical bench was continuously purged with dry air to effectively eliminate all detectable traces of atmospheric water vapor and carbon dioxide.

### Wide-Angle X-ray Scattering (WAXS)

The X-ray scattering patterns of pure α-PLL samples were obtained in a wide scattering angle range (approximately 2–160°) using a D/Max Rapid II diffractometer (Rigaku, Tokyo, Japan) equipped with a 12 kW rotating Ag anode X-ray tube, a graphite monochromator (002), and a two-dimensional curved imaging plate detector. Samples were probed in 1.5 mm diameter borosilicate glass capillaries, in transmission mode. The collimated incident X-ray beam size was 0.3 mm, and the wavelength was 0.5608 Å (Ag Kα line). All raw two-dimensional diffraction patterns were azimuthally integrated and converted to intensity versus the scattering angle (2θ) functions. The background from the empty capillary was also measured and subtracted from the patterns collected for the samples. The temperature during the thermal annealing experiments was controlled by an Oxford Cryostream Plus and a Compact Cooler, with a precision of ±5 °C. Since the scattering patterns above approximately 12°, are mostly due to the intramolecular structure of the l-lysine units, which is the same in both samples and at various thermodynamic conditions, they exhibit a similar scattering power in this range. To enable a quantitative comparison of the registered diffraction maxima, all WAXS patterns for each PLL sample were rescaled to data above approximately 12° for initial structures at room temperature.

### Differential Scanning Calorimetry (DSC)

Differential scanning calorimetry (DSC) measurements were carried out using a Mettler–Toledo heat-flux DSC equipped with a liquid-nitrogen cooling accessory and an HSS8 ceramic sensor comprising 120 thermocouples. Temperature and enthalpy calibrations were performed using certified indium and zinc standards (Mettler–Toledo). The calibration was conducted under the same experimental conditions as those used for the samples, including identical crucibles, purge gas, and flow rate, and a heating/cooling rate of 10 K/min. An empty-pan run was recorded and used for baseline correction. Temperature calibration was established based on the melting onset temperatures of the standards, while enthalpy calibration was determined by integrating their melting endotherms. The accuracy of the calibration was verified at the beginning of each measurement series.

Samples were loaded into 40 μL open aluminum crucibles and prepared outside the DSC chamber. After sealing, the crucibles were placed in the DSC cell. To remove residual moisture and any volatile solvent traces, each sample was first heated from 293 to 363 K and held isothermally for 5 min. The specimens were then cooled to 273 K and reheated to 403 K to determine the glass transition temperature and identify other thermal events. Upon reaching 403 K, the sample was isothermally annealed for 15 min, then cooled to 273 K and reheated to 403 K to assess changes in the thermal response. This sequence (cooling, reheating, and annealing) was repeated with increasing annealing times, in order to evaluate the effect of annealing on the thermal behavior of the sample. All measurements were conducted in at least duplicate to ensure the reproducibility and accuracy of the data.

### Broadband Dielectric Spectroscopy (BDS)

Dielectric measurements were performed using a Novocontrol BDS system comprising an α Impedance Analyzer, an active sample cell, and a Quatro Cryosystem temperature controller. The experiments were conducted over the temperature range of 273–403 K and a frequency range from 10^–1^ to 10^6^ Hz. All samples were analyzed using a parallel-plate capacitor geometry with a diameter of 10 mm, as described previously.^52^

Prior to the measurements, the samples were preheated outside the measuring chamber to 363 K and held for 5 min to remove residual water and any volatile solvent traces. Subsequently, following a protocol equivalent to that used in the DSC experiments, the samples were subjected to multiple heating–cooling cycles in the range 273–403 K with a step of 5 K. These cycles were combined with progressively longer isothermal annealing steps (900, 1800, 3600, and 10,800 s, respectively) at 403 K. This procedure was employed to examine the influence of thermal history and annealing duration on the dielectric response and electrical conductivity of the samples.

### Thermogravimetric Analysis (TGA)

The thermal stability of α-PLL samples was tested using a Mettler TG 50 apparatus equipped with a Mettler MT5 balance (Mettler Toledo, Switzerland).

## Results and Discussion

α-PLL is a homopeptide composed of l-lysine units arranged in a linear chain. Unlike globular proteins, this polymer lacks tertiary or quaternary structures; however, its properties can vary significantly with molecular weight. For this study, α-poly-l-lysine hydrobromide (α-PLL) samples with different molecular weights were synthesized via ring-opening polymerization (ROP) of the corresponding *N*-carboxyanhydride monomer, Lys­(Cbz)-NCA. This synthetic route ensures precise control over the molecular weight and yields low dispersity (Table 1). Following polymerization, the polymers were deprotected using a standard procedure involving a 33% solution of hydrogen bromide (HBr) in acetic acid. This process yielded macromolecules with protonated pendant amino groups. All products were characterized by proton nuclear magnetic resonance spectroscopy (^1^H NMR) and size exclusion chromatography (SEC); see the SI. The number-average molecular weights (*M*_n_) of the final deprotected α-PLL hydrobromide samples were determined by ^1^H NMR end-group analysis, including Br^–^ in the repeating-unit molar mass. Due to solubility constraints and the aggregation tendency of the unprotected polypeptide, SEC measurements were conducted on the fully carbobenzoxy-protected precursor polymers (Cbz-PLL). The SEC-derived values should therefore be regarded as apparent molecular weights and dispersities of the protected precursors, obtained using calibration against PEO standards. Thus, ^1^H NMR provides the *M*_n_ values of the final ionic materials, whereas SEC characterizes the molecular-weight distribution of their soluble protected precursors.

**Table 1:** Basic Characteristics of the Obtained α-PLL Samples (**PLL1** and **PLL2**)

sample	DP^a^	*M*_n_,_NMR_ (g/mol)^a^	*M*_n,SEC,app_ (g/mol)^b^	*Đ* ^b^
(**PLL1**)	47	9800	11,500	1.26
(**PLL2**)	70	14,600	12,400	1.29

a Determined by ^1^H NMR spectroscopy in deuterated dimethyl sulfoxide (DMSO) for deprotected α-PLL hydrobromide, including Br^–^ in the repeating-unit molar mass.

b Determined by SEC for fully Cbz-protected precursor polymers in *N*,*N*-dimethylformamide (DMF) containing 10 mM LiBr at 323 K, using calibration against PEO standards. SEC-derived *M*_n_ values should be regarded as apparent molecular weights (*M*_n,SEC,app_).

The synthesized polymers were first subjected to thermogravimetric analysis (TGA), which provided two essential pieces of information for the subsequent experimental work. First, both α-PLL samples were found to be thermally stable up to approximately 450 K, excluding polymer decomposition within the temperature range relevant to this study (Figure 1). Second, TGA identified the temperature range and magnitude of residual water loss (2–4% wt. at 360 K), which was crucial for optimizing sample preparation and defining the thermal treatment protocol. This was particularly important because even small amounts of moisture can plasticize polypeptide matrices and modify their molecular mobility,^53^ potentially distorting the experimental results.

**Figure 1:**
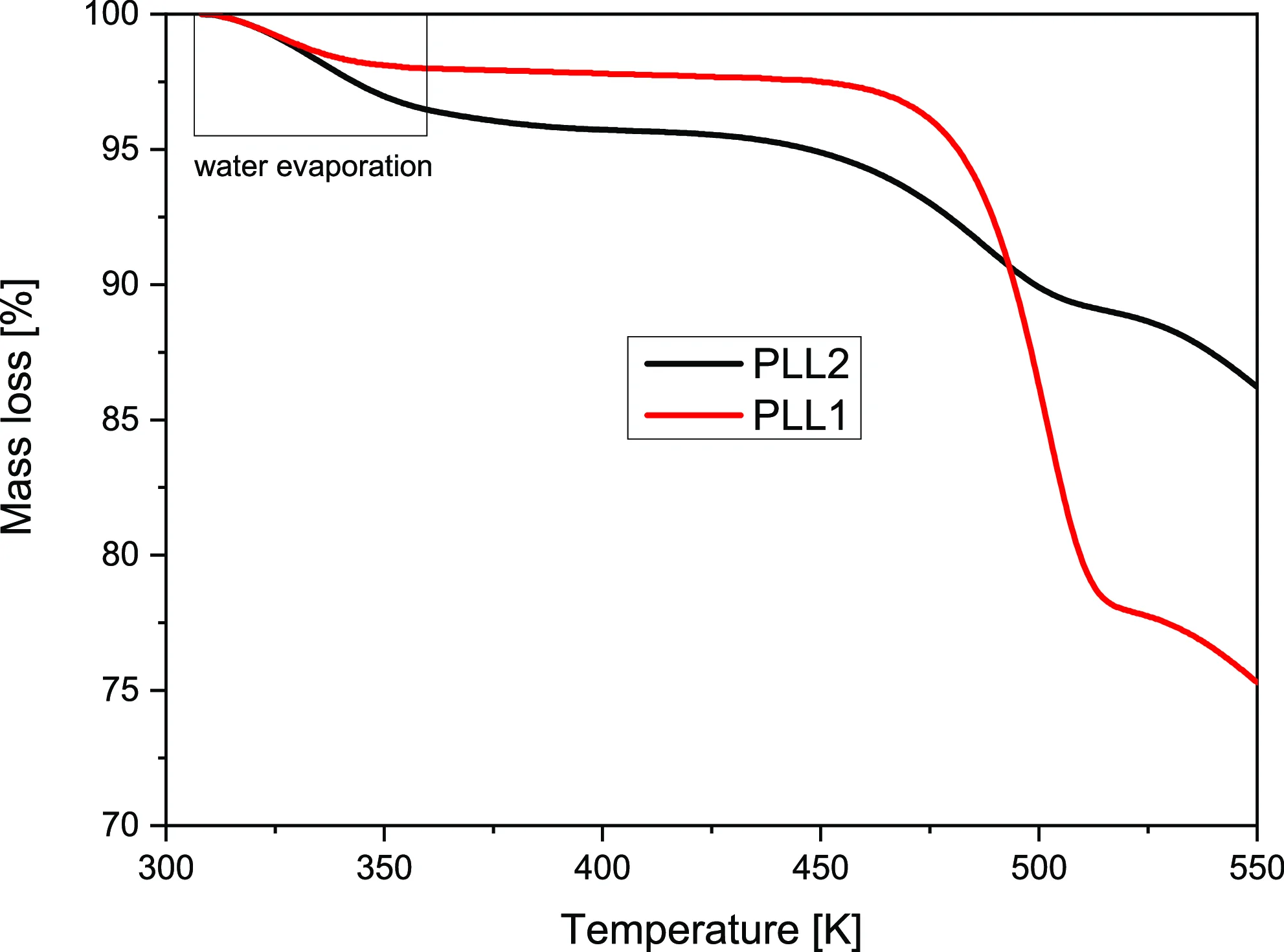
Results of thermogravimetric analysis of **PLL1** and **PLL2** with the water evaporation until 360 K highlighted in the rectangle.

Based on the TGA results, FT-IR spectroscopy was first employed to assess the influence of residual water on the vibrational response of the investigated α-PLL samples. The as-prepared **PLL1** and **PLL2** samples, recovered directly after freeze-drying, were initially measured at room temperature (Figure S4). Subsequently, the same materials were heated above the temperature range in which TGA indicated the removal of loosely bound water and then cooled back to room temperature. This protocol enabled the identification of absorption bands associated with residual water, the assessment of water-removal-induced spectral changes, and the verification that no clear spectroscopic contribution from loosely bound/free water remained after heating above 373 K (Figure S4). The most pronounced dehydration-induced changes were observed in the 3600–3000 cm^–1^ region, where the broad O–H stretching contribution of residual water, partly overlapping with N–H stretching vibrations, markedly decreased. Noticeable variations were also visible in the amide I region (1750–1570 cm^–1^), where the bending vibration of molecular water overlaps with peptide carbonyl vibrations. Reducing this water contribution, therefore improved the separation of the conformationally sensitive amide I components. It should be noted that this dehydration protocol refers primarily to the removal of loosely bound/free water, as indicated by TGA and by the decrease of the broad O–H stretching contribution in the FT-IR spectra, rather than to the production of an absolutely water-free material. A small amount of tightly bound residual water may still remain associated with ionic and peptide groups in α-PLL hydrobromide. Nevertheless, more aggressive drying aimed at removing this strongly bound fraction would require higher temperatures and could induce additional irreversible changes or thermal degradation of the α-PLL hydrobromide matrix. Accordingly, the dehydration conditions were selected to minimize the contribution of residual water while avoiding thermally induced degradation of the polymer. Importantly, the TGA curve shows no distinct additional mass-loss step immediately above the temperature range associated with the removal of loosely bound water. Therefore, although a minor contribution from strongly bound residual water cannot be completely excluded, water evaporation is unlikely to be the primary factor responsible for the changes observed in the amide I region discussed below.

The room-temperature FT-IR spectra provided the basis for a comprehensive vibrational assignment of both polylysines. The detailed band assignments to the vibrations of the corresponding functional groups are presented in the SI, Figure S5. This preliminary analysis, benchmarked against literature data, enabled us to identify the key amide I markers of the secondary structures present in **PLL1** and **PLL2**. In particular, the bands at approximately 1624, 1650, and 1670 cm^–1^ were assigned to β-sheet, α-helical, and β-turn/antiparallel β-sheet conformations, respectively. The identification of these bands was essential for interpreting the subsequent temperature- and time-dependent FT-IR measurements, as they served as direct spectral markers of conformational reorganization. It should also be mentioned that, according to the literature, the asymmetric bending vibrations of protonated side-chain amino groups (−NH_3_^+^) typically occur in the 1630–1620 cm^–1^ region with low integral intensity.^54–56^ Given that the well-resolved β-sheet peaks strongly dominate the 1640–1600 cm^–1^ range, any background contribution from these side-chain modes to the total integrated area is expected to be minor compared with the dominant β-sheet contribution and should not alter the observed kinetic trends. Therefore, the integrated intensity in the 1640–1600 cm^–1^ region may serve as a suitable apparent β-sheet-related spectral marker rather than as an absolute quantitative measure of β-sheet content.

Analysis of the amide I region revealed clear differences between the two samples. For the lower-molecular-weight polymer, **PLL1**, the contribution of β-sheet conformations was markedly larger, as evidenced by the pronounced intensity of the band at 1624 cm^–1^. In contrast, **PLL2** showed a stronger contribution from the α-helical component near 1650 cm^–1^, indicating that the two polylysines differ substantially in their dominant secondary structure.

Additional structural information was obtained from the high-wavenumber region, where the N–H stretching band appeared at different positions for the two polylysines. In **PLL1**, this band was observed at 3272 cm^–1^, whereas in **PLL2** it was shifted to a higher wavenumber, 3281 cm^–1^. Since stronger hydrogen bonds (H-bonds) weaken the N–H bond and shift its stretching vibration toward lower wavenumbers, the lower position of this band in **PLL1** indicates stronger N–H···O=C H-bonding than in **PLL2**. This result is particularly important when considered together with the amide I analysis. The sample with the larger β-sheet contribution, **PLL1**, also exhibits stronger H-bonding, whereas the predominantly α-helical **PLL2** is characterized by comparatively weaker N–H···O=C interactions. This suggests that, in the investigated α-PLL samples, β-sheet-rich organization is associated with a stronger H-bonding network than the α-helical arrangement. Such behavior can be rationalized by the geometry of β-sheet structures, in which adjacent peptide chains are arranged in an extended, nearly parallel or antiparallel manner, allowing more favorable packing and more linear interchain N–H···O=C H-bonds. By contrast, in α-helical conformations, H-bonds are mainly intrachain and constrained by the helical geometry, which may lead to less optimal bond directionality and weaker effective H-bonding interactions. Thus, the N–H stretching region not only supports the amide I assignment but also shows that the β-sheet-rich **PLL1** structure is stabilized by stronger H-bonding than the predominantly α-helical **PLL2** structure.

Importantly, dehydration did not erase the characteristic amide I bands of the secondary structures already present in the freeze-dried samples. Instead, it mainly modified their relative intensities. This indicates that heating above 373 K removes loosely bound water and induces a partial redistribution of conformational populations rather than a complete conformational transformation. In **PLL1**, the relative contribution of the β-sheet-related component becomes more pronounced after dehydration, whereas bands associated with less ordered or turn-like conformations are reduced or become less resolved. In **PLL2**, the α-helical band remains clearly visible, but dehydration and further heating enhance the spectral contribution of β-sheet-like structures. Therefore, the heating step shifts the equilibrium between coexisting conformers, most likely by removing water molecules that stabilize specific H-bonding arrangements, yet both polylysines retain a mixed secondary-structure character after dehydration. It should also be noted that the relative intensities of the characteristic amide I bands indicate that the populations of these conformers differ markedly between the shorter and longer polylysine samples, i.e., both after freeze-drying and after heating: in **PLL1**, the β-sheet form remains dominant, whereas in **PLL2**, the α-helical form is the prevailing conformation (Figure S6 in the SI). This difference may reflect the different molecular weights of **PLL1** and **PLL2**, which may influence chain packing, H-bonding organization, and the ability of the chains to adopt specific secondary structures during freeze-drying and subsequent heating.

In the next step, temperature-dependent FT-IR measurements were performed to follow the conformational evolution of both α-PLL samples upon heating. For clarity, **PLL1** and **PLL2** are discussed together, since both systems undergo the same general transformation from initially mixed conformational states toward β-sheet-rich structures (Figure S6). Representative spectra recorded during heating are shown in Figure 2, while additional spectral data are provided in Figure S6. Upon increasing temperature, the most pronounced changes occurred in the amide I region, where the bands associated with α-helical and β-turn/antiparallel conformations progressively decreased in relative intensity, while the low-wavenumber β-sheet component became increasingly dominant. For **PLL1**, this process was reflected by the growth of the band at approximately 1624 cm^–1^, whereas for **PLL2**, a sharp β-sheet band developed near 1627 cm^–1^. At the same time, the band at 1650 cm^–1^, assigned to α-helical conformations, decreased markedly, especially at temperatures above the range where water loss was detected by TGA. These spectral changes demonstrate that heating promotes the reorganization of α-PLL chains from mixed conformational states toward more extended, intermolecularly associated β-sheet structures. This transformation was accompanied by systematic changes in other spectral regions. The amide II band (1575 and 1480 cm^–1^) shifted toward lower wavenumbers during heating, indicating modifications in the environment of N–H bending and C–N stretching vibrations as the β-sheet structure developed. In the high-wavenumber region, the N–H stretching band shifted slightly to higher wavenumbers, consistent with changes in the H-bonding network during dehydration and conformational reorganization. Additional broadening and red-shifting of bands in the fingerprint region, including skeletal and side-chain vibrations, further indicate changes in the local environment of the lysine side chains and ε-amino groups. Overall, the temperature-dependent FT-IR spectra indicate that heating above the water-removal range identified by TGA induces a clear, progressive conformational reorganization in both α-PLL samples. Although **PLL1** and **PLL2** differ in their initial conformer populations after freeze-drying, both samples evolve toward β-sheet-rich structures at elevated temperature. This confirms that the thermally induced transformation is not merely a dehydration effect, but involves a substantial rearrangement of the polypeptide secondary structure and the development of a more organized intermolecular H-bonded network.

**Figure 2:**
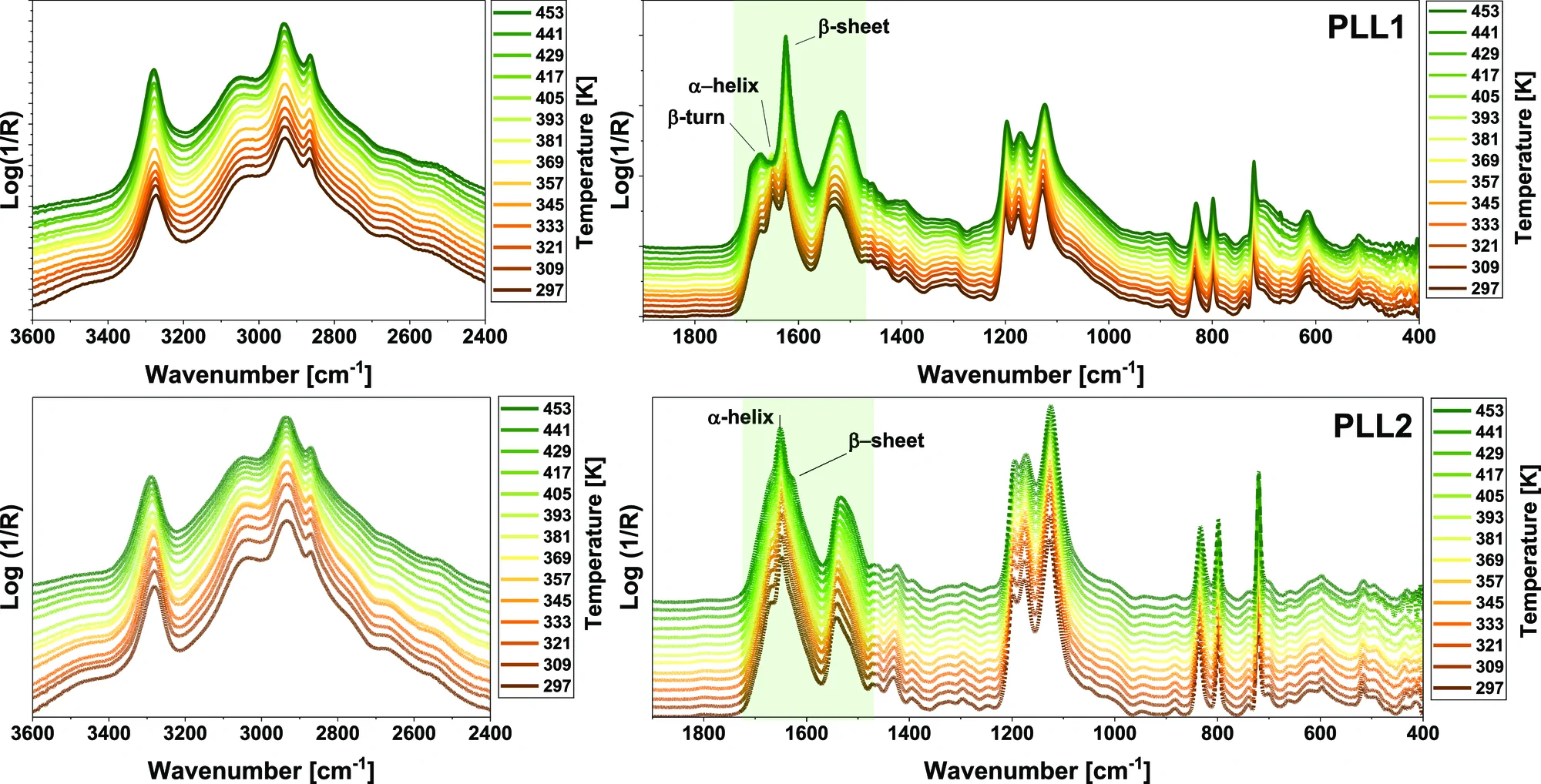
Temperature-dependent FT-IR spectra of **PLL1** (upper panels) and **PLL2** (bottom panels) measured in the wavenumber ranges: 3600–2400 and 1900–400 cm^–1^.

Having characterized the temperature-induced response of both α-PLL samples and the gradual reorganization of their H-bonding network following the dehydration event detected by TGA at approximately 353 K, we next focused on the kinetics of this conformational transformation. Since **PLL1** and **PLL2** exhibited qualitatively similar temperature-dependent spectral evolution, the full kinetic analysis was performed for **PLL2** over a broad temperature range. To this end, time-dependent FT-IR spectra of **PLL2** were recorded under isothermal conditions at selected temperatures, namely 383, 393, 403, 413, 433, and 453 K. Representative spectra collected at 393 and 413 K are shown in Figure 3, while other data are presented in Figure S7 in the SI. For each temperature, the integrated intensity of the amide I component in the 1640–1600 cm^–1^ region, assigned to β-sheet conformations, was determined and plotted as a function of time (Figure 4a). This analysis allowed us to monitor the growth of the β-sheet population and to quantify the kinetics of the structural transition. The resulting kinetic curves clearly show that the time required to reach structural saturation decreases strongly with increasing temperature. At 383 K, the β-sheet signal continued to evolve for more than 540 min, whereas at 453 K saturation was reached within approximately 60 min. This pronounced acceleration demonstrates that the conformational reorganization is strongly thermally activated. The growth curves were fitted using a single-exponential growth function as an apparent kinetic description, from which the apparent rate constants *k*, were determined for each temperature. This fitting approach was applied consistently to all temperatures to enable comparison of the transformation rates, and the extracted *k* values should therefore be regarded as apparent kinetic parameters. The temperature dependence of these rate constants was then analyzed using the Arrhenius equation:

1 $$\ln\left(k\right)=\ln\left(A\right)-\frac{E_{A}}{\mathrm{RT}}$$

**Figure 3:**
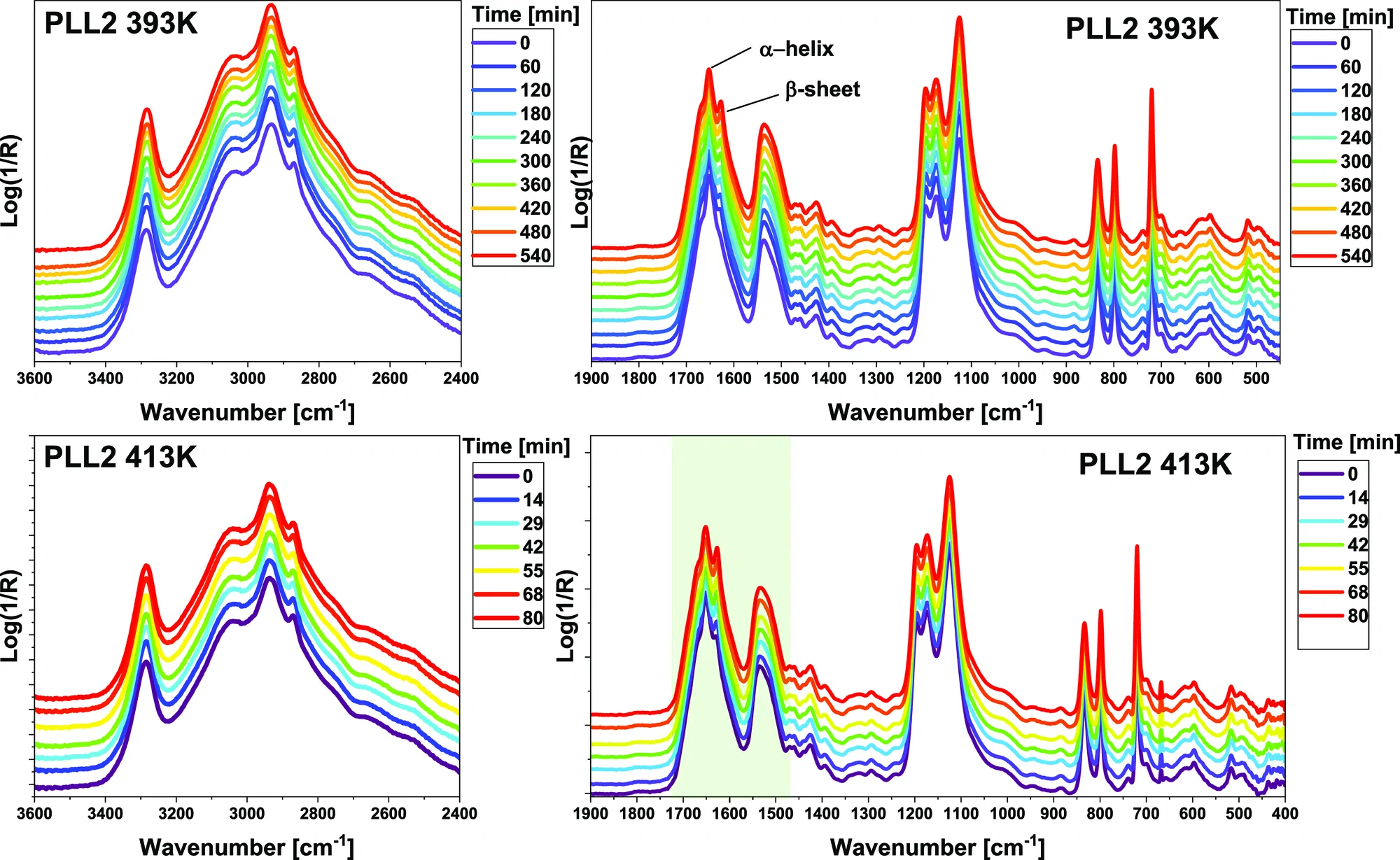
Time-dependent FT-IR spectra of **PLL2** measured at 393 K (upper panels) and 413 K (bottom panels) in the wavenumber ranges: 3600–2400 and 1900–400 cm^–1^.

**Figure 4:**
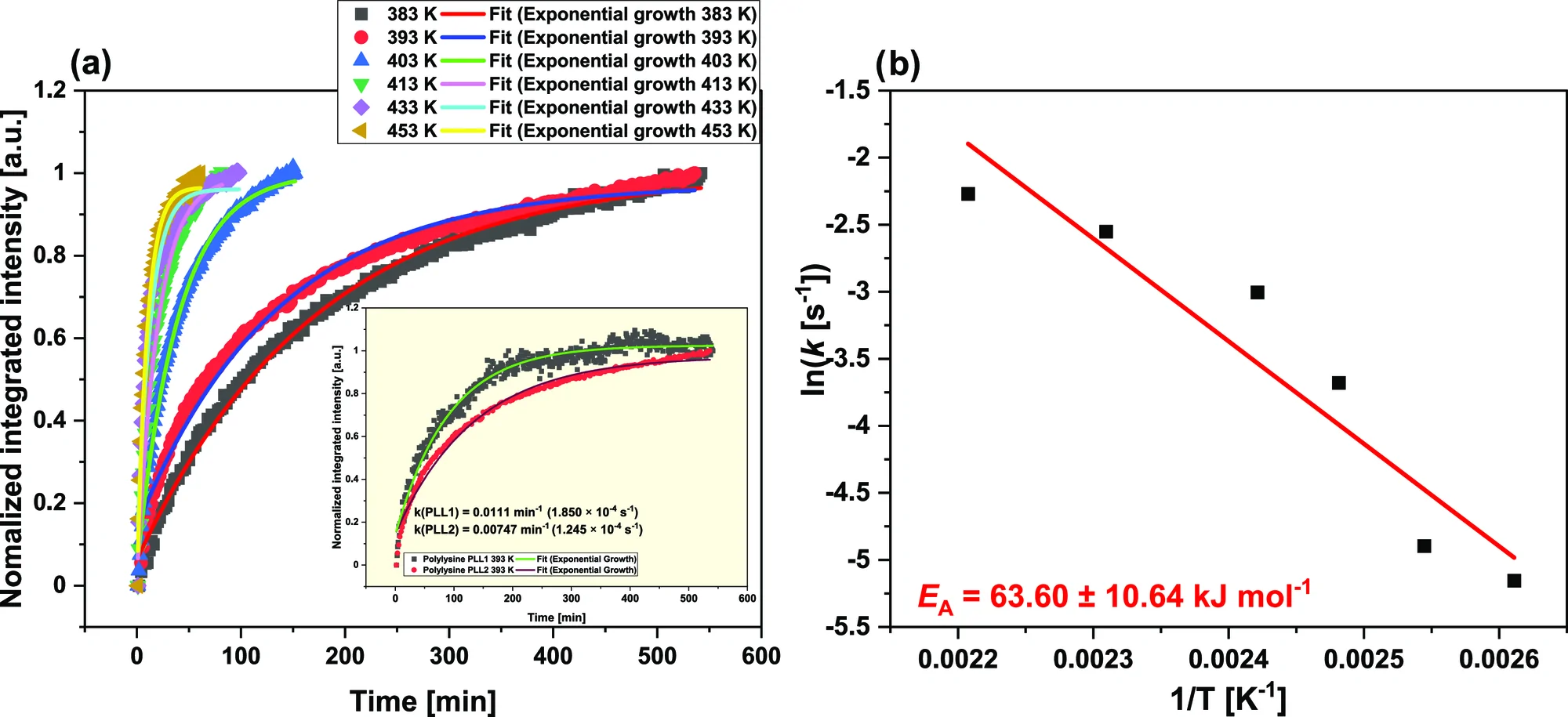
Temperature-dependent kinetics of the structural transition in PLL samples. (a): Normalized integrated intensity of the β-sheet band as a function of time at selected temperatures (383, 393, 403, 413, 433, and 453 K) for **PLL2**. Solid lines represent fits using an exponential growth function. The inset highlights a comparison between two PLL samples with different chain lengths at 393 K, including their respective rate constants (*k*). (b): Arrhenius plot showing the linear fit of ln­(*k*) versus 1/*T* for **PLL2**. The calculated apparent activation energy for the conformational reorganization is *E*_A_ = 63.6 ± 10.6 kJ mol^–1^.

where: *A* is the pre-exponential factor, *E*_A_ is the activation energy, *R* is the gas constant, and *T* is the absolute temperature. The natural logarithms of the rate constants were presented as a function of 1/*T*, and the activation energy was obtained from the slope of the resulting Arrhenius plot (Figure 4b). The calculated value of *E*_A_ was approximately 64 kJ mol^–1^, providing a quantitative measure of the energy barrier associated with the reorganization of the polypeptide backbone into a stable β-sheet architecture. This value is consistent with the energy range typically associated with cooperative breaking and reorganization of multiple H-bonds during protein secondary-structure transitions.^57,58^ Moreover, it falls within the expected range for solid-state transformations of polypeptides. This suggests that, after the initial dehydration step occurring near 353 K, further heating primarily drives backbone reorganization and the development of intermolecular β-sheet structures, rather than being dominated by solvent-related effects.

To compare the kinetics of conformational reorganization in both α-PLL samples and to evaluate the possible influence of chain length on the rate of β-sheet formation, additional time-dependent FT-IR measurements were performed for **PLL1** at 393 K (Figure S8). The integrated intensity of the amide I band in the 1640–1600 cm^–1^ range was analyzed in the same way as for **PLL2**, allowing a direct comparison of the reorganization rates (Figure 4a). The exponential fit yielded apparent rate constants of the same order of magnitude for **PLL1** and **PLL2** at the same temperature, with values of *k* = 1.850 × 10^–4^ s^–1^ and *k* = 1.245 × 10^–4^ s^–1^, respectively (see the inset). Thus, although the overall rate of β-sheet formation is comparable in both systems, the slightly lower rate constant obtained for **PLL2** indicates that the conformational transformation proceeds more slowly in the higher-molecular-weight polymer. This tendency is consistent with the expected slower conformational reorganization of longer polypeptide chains, for which segmental rearrangements and the formation of an ordered β-sheet network may require longer times. Further kinetic measurements for **PLL1** at other temperatures were not performed since there was much higher population of β-sheet conformation in this polymer. Thus the analysis of the data would be much more difficult to follow because of weak changes in the amplitude of band observed at 1640–1600 cm^–1^.

The fingerprints of thermally induced conformational reorganization toward β-sheet-rich structures in α-PLL samples were further confirmed by wide-angle X-ray scattering (WAXS) analysis. The scattering patterns of the synthesized **PLL1** and **PLL2** samples, collected at 293 K after the dehydration process (dark blue curves in the upper panels of Figure 5), exhibit a series of small-angle reflections that are a hallmark of 2D hexagonal symmetry. The ratios of their positions of ∼1:√3:2 are consistent with the (10), (11), and (20) planes of the liquid-crystalline-like 2D hexagonal lattice. Previous studies have reported the formation of a similar bidimensional hexagonal structure of rods in various **PLL** systems, in which the rods contain peptide chains in an α-helix conformation.^59–62^ The position of the (10) reflection can be taken for the estimation of the interplanar spacing, *d*_(10)_, and the hexagonal lattice constant (*a*), according to the formula: $a=\sqrt{4/3}{\cdot d}_{\left(10\right)}$ , where $d_{\left(10\right)}=\frac{\lambda }{2\sin\theta_{\left(10\right)}}$ . Here, *d*_(10)_ ≈ 12.5 and 13.0 Å, while *a* ≈ 14.5 and 15.0 Å for **PLL1** and **PLL2**, respectively. Briefly, since the *a* parameter describes the lateral spacing within the hexagonal arrangement of α-helical polylysine chains, it is equivalent to the center-to-center distance between two nearest-neighbor chains; the slight difference in the *a* parameter between **PLL1** and **PLL2** highlights differences in the packing of their chains–they are less densely packed in **PLL2**. However, the most pronounced difference between the two samples is not the small change in the lattice constant, but the degree of development of the hexagonal reflections. The diffraction peaks associated with the hexagonal ordering of polymer chains are sharper and more clearly resolved for **PLL2**, whereas in **PLL1** they are much broader and weaker. Moreover, the reflection (20) is hardly distinguishable. This indicates a higher degree of hexagonal order of α-helical chains in **PLL2**. Such behavior is consistent with the higher molecular weight of **PLL2**, since longer polylysine chains are generally more capable of stabilizing α-helical conformations.^63^ Simultaneously, the weaker hexagonal order in **PLL1** is also related to a larger β-sheet population and reduced α-helical contribution in this sample. In other words, the β-sheet-rich fraction in **PLL1** reduces the liquid-crystalline-like hexagonal packing, leading to broader and less developed diffraction reflections in the small-angle range of the WAXS pattern. Thus, the WAXS results are consistent with the FT-IR analysis: **PLL2** preserves a more ordered hexagonal organization of α-helical chains, whereas **PLL1** contains a larger β-sheet structure contribution and suppresses the hexagonal order.

**Figure 5:**
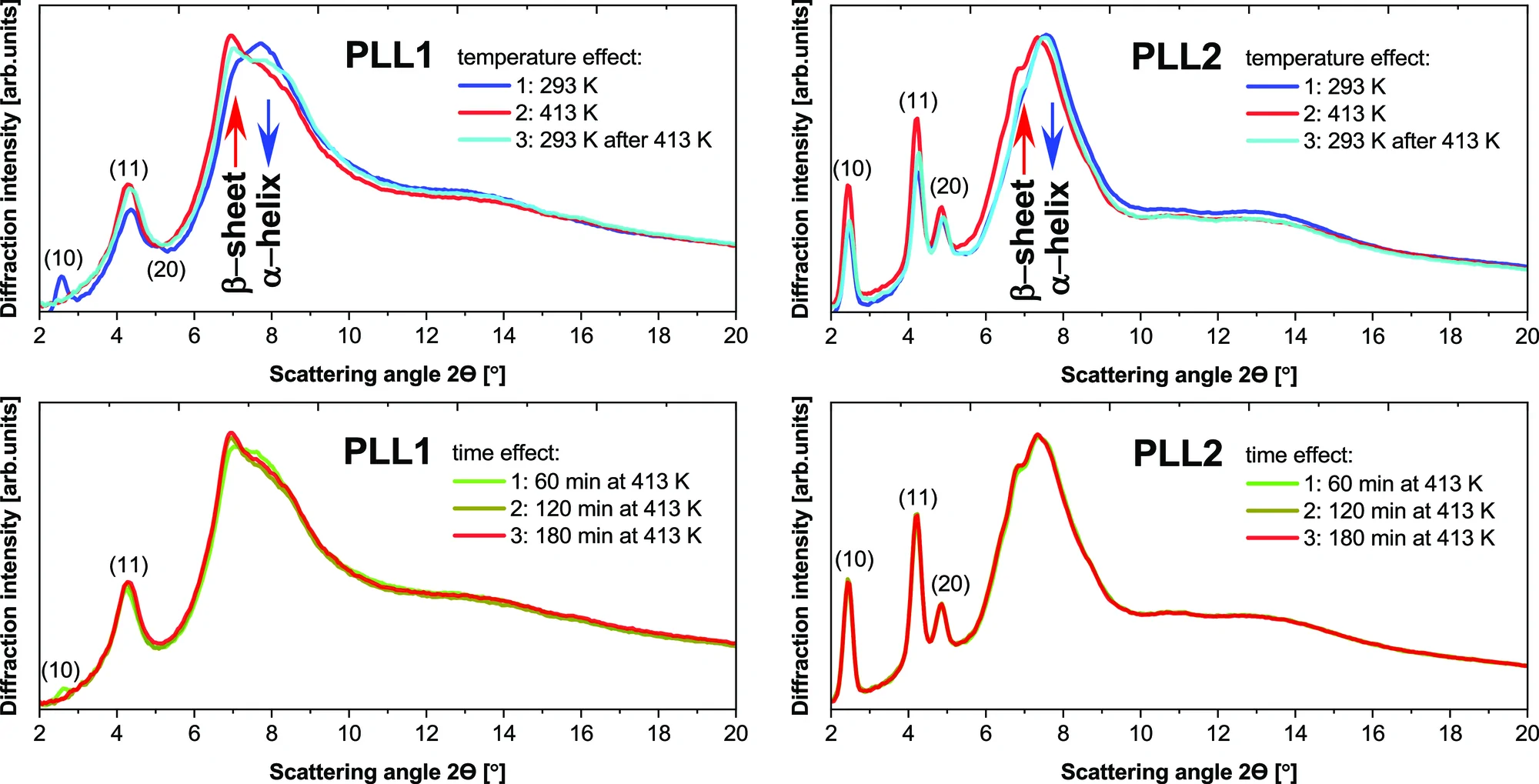
X-ray diffraction patterns of **PLL1** (left) and **PLL2** (right) measured as a function of temperature (upper) and time at 413 K (bottom).

Besides the small-angle reflections, the XRD patterns of the examined samples exhibit a broad, intense signal in the wider-angle range, consisting of two components, with the second component, at wider 2θ angles ∼8°, more pronounced at 293 K. The position of this diffraction feature corresponds to a distance of ∼4 Å, which represents the average spacing between loosely packed polylysine chains and their flexible aliphatic side groups as well as the distance between adjacent turns of the α-helix. However, a contribution from structural correlations along the β-strand and a secondary, diffuse equatorial reflection due to packing of β-sheets may contribute to the diffraction data in this range as well.^64^ The lower-angle component of the broad halo, at 2θ ∼ 7°, arises from the lateral spacing of approximately 4.6 Å, which is close to previous literature observations for the distance between adjacent β-strands within the same sheet.^65^ Therefore, it is strong experimental evidence of the β-sheet organization. This is consistent with the results of FT-IR studies, indicating that both polypeptides comprise a mixture of α-helix and β-sheet conformations. In line with the findings of infrared investigations, and as shown in the upper panels of Figure 5, the relative amplitude of the first component at ∼7°, as compared to the second peak at ∼8°, increases upon heating to 413 K, demonstrating a thermally induced formation of β-sheet-rich structures in both α-PLL samples. Moreover, in the case of **PLL1**, the (10) and (20) reflections diminish, whereas the reflection around ∼4° becomes more intense after the heat treatment. This feature may be partially due to a distorted hexagonal lattice, but it may also contain a contribution from the lamellar organization of emerging β-sheets with an intersheet spacing of ∼7.5 Å. In the case of **PLL2**, all (10), (11), and (20) reflections get sharper and more intense at higher temperatures, so the liquid-crystalline hexagonal order is not suppressed, in contrast to the **PLL1** system. The WAXS data collected after subsequent cooling of these systems down to 293 K (cyan curves in the upper panels of Figure 5) also demonstrate that the structures of these PLL systems do not return to their initial states before the heating cycle. The hexagonal structure, and thus the associated diffraction reflections in **PLL1**, are not rebuilt, while in **PLL2**, shrinking of the hexagonal lattice is observed after the thermal annealing, since the associated reflections are shifted toward higher scattering angles, which results in a slight decrease in the lattice parameter (*a* ≈ 14.8 Å after thermal annealing versus *a* ≈ 15 Å before annealing). This behavior can be attributed to slight compaction of the interchain structure. In other words, thermal annealing provides the energy needed for polylysine chains to adopt more stable conformations that pack more tightly.

Furthermore, WAXS was used to monitor the time frame of the structural transformation in both α-PLL polypeptides at constant temperature. The lower panels in Figure 5 show changes in the XRD patterns after heating for 60, 120, and 180 min at 413 K. As demonstrated, this process requires around 120 min for **PLL1** and **PLL2** at 413 K. After this time, no further changes in the diffraction patterns as a function of time were observed. Importantly, the time-resolved FT-IR analysis indicates a similar time scale for the stabilization of the β-sheet-related structural reorganization, as shown in Figure 4(a). At 413 K (green curve), the normalized integrated intensity of the β-sheet-related IR contribution approaches saturation within the first ca. 100 min, demonstrating good agreement between the molecular-level changes detected by FT-IR and the longer-range structural rearrangements monitored by WAXS. A more detailed kinetic analysis using WAXS is, however, not feasible because of the markedly different temporal resolutions of the two techniques. An FT-IR spectrum accumulated from 16 scans is acquired in approximately 20 s, whereas the collection of a single WAXS diffractogram requires ca. 1 h to achieve sufficient counting statistics. Therefore, although WAXS confirms the occurrence of longer-range packing changes on a comparable time scale, its lower temporal resolution makes FT-IR better suited to the quantitative kinetic analysis of the α-to-β structural transformation.

Having described the thermally induced conformational change in both α-PLL samples, we next examined how this process affects their thermal properties and glass transition temperature (*T*_g_). As shown in Figure 6a,b, the initial **PLL1** and **PLL2** samples exhibit relatively high and comparable *T*_g_ values of 345 and 341 K, respectively. Interestingly, **PLL2**, the higher-molecular-weight sample, shows a slightly lower *T*_g_, which may appear counterintuitive at first glance. However, this apparent discrepancy can be rationalized in light of the FT-IR results for the freeze-dried samples and those recovered after subsequent thermal treatment. These measurements demonstrated that both α-PLL samples initially contain different populations of β-sheet, α-helical, and β-turn/antiparallel β-sheet conformations. Therefore, the initial *T*_g_ values should not be interpreted solely in terms of molecular weight, but rather as a consequence of both chain length and conformational composition.

**Figure 6:**
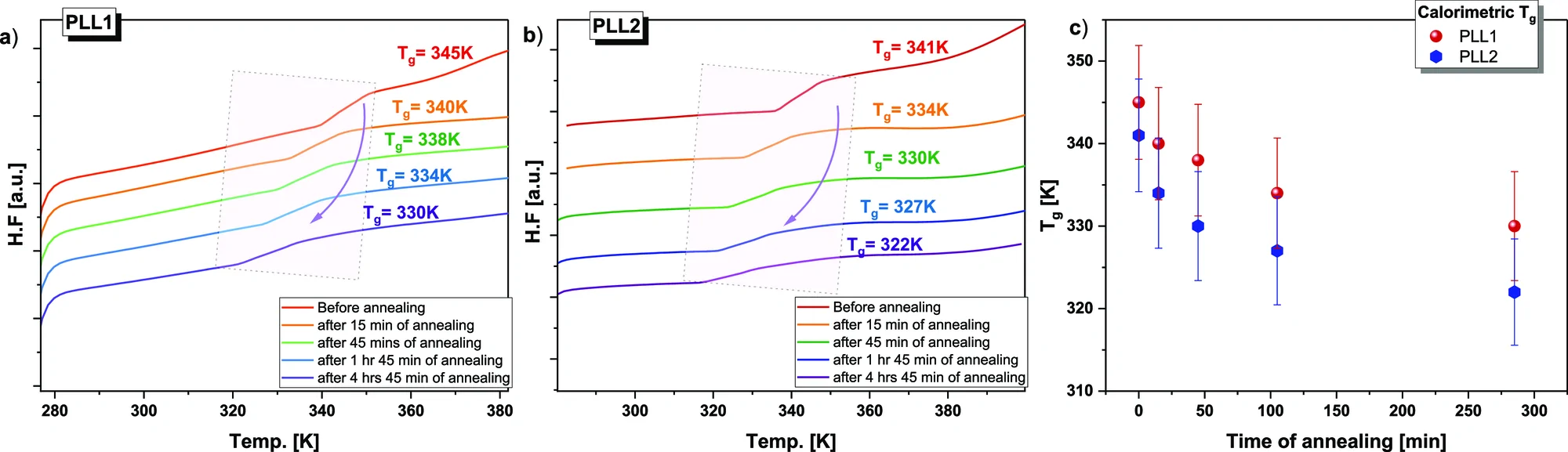
DSC thermograms of **PLL1** (a) and **PLL2** (b) measured at a heating rate of 10 K/min, with the corresponding glass transition temperatures (*T*_g_) indicated. Panel (c) shows the calorimetric *T*_g_ values as a function of annealing time for **PLL1** and **PLL2**. Error bars represent standard deviation (SD) from repeated measurements.

Nevertheless, to verify whether the conformational reorganization affects segmental mobility, both samples were subjected to a systematic annealing protocol. The samples were annealed at *T* = 403 K for selected time intervals, then cooled well below *T*_g_ and reheated to *T* = 403 K. This procedure was repeated until no further changes in *T*_g_ were observed. Representative DSC heating curves recorded after different annealing times are shown in Figure 6a,b. In each thermogram, the glass transition is clearly visible as a heat-capacity step that progressively shifts toward lower temperatures with increasing annealing time. Upon annealing, *T*_g_ decreases from 345 K to approximately 330 K for **PLL1** and from 341 K to approximately 322 K for **PLL2** after prolonged annealing. Thus, the thermally induced conformational reorganization leads to a *T*_g_ depression of about 15–20 K. The time evolution of *T*_g_ is summarized in Figure 6c. For both samples, the reduction in *T*_g_ is most pronounced during the initial stage of annealing, particularly within the first 50–100 min, followed by a much slower evolution at longer times. Although the DSC traces may include baseline curvature and weak enthalpy-relaxation contributions, *T*_g_ was determined consistently from the midpoint of the heat-capacity step using the same baseline procedure for all annealing stages. Therefore, the absolute *T*_g_ values should be interpreted with the uncertainty typical of glass-transition analysis in weakly relaxing polymeric systems. Nevertheless, the observed *T*_g_ decrease is systematic, time-dependent, and occurs in parallel with the β-sheet-related spectral changes detected by FT-IR and the mesomorphic packing reorganization observed by WAXS. Thus, minor baseline and enthalpy-relaxation effects cannot be fully excluded, but they are unlikely to account for the overall annealing-induced decrease in *T*_g._ More specifically, the FT-IR kinetic data (Figure 4a) show that the growth of the β-sheet component proceeds rapidly at the beginning of the isothermal treatment and then gradually approaches saturation. The observed decrease in *T*_g_ indicates that the thermally induced formation of β-sheet-rich structures modifies the packing and mobility of the α-PLL chains. This result is particularly intriguing because, in many polymeric systems, an increase in structural order and strengthening of intermolecular H-bonding interactions would typically be expected to increase *T*_g_.^66,67^ However, α-PLL does not behave as a conventional amorphous polymer. Annealing induces a conformational transformation toward a β-sheet-rich structure in both PLL samples, while the accompanying changes in liquid-crystalline order differ markedly between the two polymers and depend on their molecular weight. This distinction is important for interpreting the observed decrease in *T*_g_. Although thermally induced mesophase reorganization has been reported to cause internal plasticization and a reduction in *T*_g_ in selected liquid-crystalline polymers,^68–71^ such an interpretation appears less likely in the present case. Specifically, the two PLL samples exhibit different changes in liquid-crystalline organization upon annealing, whereas both show a comparable decrease in *T*_g_ and, most importantly, both undergo conformational reorganization toward β-sheet-rich structures. Therefore, the formation of β-sheets, rather than the sample-specific changes in liquid-crystalline order, appears to be the common structural factor underlying the *T*_g_ depression detected by DSC. We hypothesize that the lower *T*_g_ of the β-sheet-rich state results from enhanced segmental mobility associated with a redistribution of intermolecular and intramolecular hydrogen-bonding constraints during the α-to-β transformation.

It is also worth noting that changes in *T*_g_ associated with subtle molecular rearrangements have been reported for small-molecule glass formers, including saccharides and glibenclamide.^72,73^ In those systems, mutarotation or tautomerization processes were monitored by dielectric spectroscopy through changes in structural relaxation dynamics and glass-transition-related behavior. However, in contrast to the response observed here for α-PLL, those molecular transformations were accompanied by an increase in *T*_g_. This comparison further highlights the unusual nature of the conformational reorganization in α-PLL, in which the development of β-sheet-rich structures is accompanied by a decrease, rather than an increase, in the glass transition temperature. Our observation also helps clarify the apparent discrepancy with the conclusions of Papadopoulos et al. regarding the proposed independence of glass-transition dynamics from secondary structure.^42^ These authors investigated carbobenzoxy-protected poly-l-lysine (Cbz-PLL), in which the amino groups of the side chains were blocked by bulky, rigid, and hydrophobic aromatic Cbz moieties. Such substituents strongly affect intermolecular packing and may substantially restrict the conformational freedom of both the side chains and the polypeptide backbone. In particular, the steric constraints imposed by the densely distributed protecting groups may hinder, or even prevent, extensive structural rearrangement between α-helical and β-sheet conformations in the solid state. Consequently, the absence of a measurable dependence of *T*_g_ on secondary structure in the study of Papadopoulos et al. may result from the limited ability of protected Cbz-PLL to undergo a substantial α-helix-to-β-sheet transformation under the applied experimental conditions. In contrast, the α-PLL hydrobromide investigated here is a fully deprotected ionic polypeptide, in which the side chains are present as protonated NH_3_^+^ groups charge-balanced by small Br^–^ counterions. The absence of bulky protecting groups leaves the polypeptide backbone considerably more accessible to conformational reorganization. Accordingly, the transformation between α-helical and β-sheet-rich structures can proceed much more readily in the deprotected material. In the present samples, the occurrence of this transformation is supported by the combined FT-IR and WAXS results, which demonstrate a thermally induced increase in the β-sheet contribution. This structural evolution is accompanied by a downward shift in *T*_g_, indicating that the glass-transition behavior of deprotected α-PLL is sensitive to changes in secondary structure. Therefore, the apparently different conclusions of the two studies are not necessarily contradictory, but rather reflect the fundamentally different conformational accessibility of protected and deprotected PLL.

Following the DSC analysis and the demonstration that annealing affects the glass transition temperature of α-PLL, BDS was employed to gain further insight into the dielectric response of the investigated systems during thermal treatment. Owing to the presence of charged groups in α-poly-l-lysine hydrobromide, both samples are expected to exhibit a pronounced conductivity contribution. Moreover, changes in charge transport may accompany the thermally induced conformational reorganization toward β-sheet-rich structures. For this reason, the dielectric analysis was focused primarily on the evolution of the real part of the complex electric conductivity, σ′(f), as a function of annealing time.

As described in Materials and Methods section, the samples were measured during successive heating cycles after isothermal annealing at 403 K for 900, 1800, 3600, and 10,800 s, corresponding to a cumulative annealing time of 17,100 s. This protocol was selected to ensure consistency with the DSC experiments. Representative conductivity spectra recorded for the investigated samples are shown in Figure 7. Over the broad frequency range from 10^–1^ to 10^6^ Hz, σ′(f) exhibits a complex frequency response, including a low-frequency decrease caused by electrode polarization effects, a plateau/shoulder region, a step-like contribution associated with Maxwell–Wagner–Sillars (MWS) polarization, and a high-frequency power-law dispersion. The MWS contribution is most likely related to the electrically heterogeneous nature of the compacted powder samples, where grain boundaries can hinder long-range charge transport and contribute to interfacial polarization.^74,75^ Hence, the higher-frequency conductivity plateau can be attributed to short-range charge transport within individual grains, whereas the lower-frequency plateau reflects long-range charge transport across the sample, including the contribution of grain boundaries. Therefore, the latter plateau-like region (10–100 Hz) was used to determine the apparent dc conductivity, σ_DC,app_, of the material. The lowest-frequency region affected by electrode polarization was excluded from the analysis. For both **PLL1** and **PLL2**, σ′ decreases systematically with increasing annealing time over nearly the entire investigated frequency range. This indicates a progressive reduction in charge transport within the polylysine matrix during thermal treatment. **PLL2** exhibits lower absolute conductivity values across the measured frequency window, whereas the relative change in conductivity upon annealing is more pronounced for the lower-molecular-weight sample, **PLL1**. This behavior is consistent with molecular-weight-dependent transport, since higher molecular weight generally reduces segmental mobility and can affect ion-transport pathways in polymeric conductors. Moreover, α-PLL has previously been reported to exhibit molecular-weight-dependent dielectric and conductometric responses.^76,77^ The progressive suppression of conductivity upon annealing indicates that charge transport in α-poly-l-lysine hydrobromide is sensitive to structural changes occurring during thermal treatment. This behavior is not straightforward, because DSC shows a simultaneous decrease in *T*_g_, which would normally suggest enhanced segmental mobility and could, in principle, favor charge transport. Therefore, the decrease in the frequency-dependent real part of the complex conductivity, σ′(f), cannot be explained solely in terms of glass-transition-related dynamics. The reduction in dc conductivity upon annealing is unlikely to arise from a reduction in the number of Br^–^ charge carriers, because TGA confirms that the polymer remains chemically stable under the applied thermal conditions. According to the formula σ_DC_ = *nq*μ, where *n* is the concentration of mobile charge carriers, *q* is their charge, and μ is their mobility, the observed decrease should therefore be attributed primarily to reduced effective ion mobility in the β-sheet-rich state. This interpretation is supported by Chen et al.,^78^ who demonstrated that α-helical polypeptides exhibit markedly higher ionic conductivity than their random-coil analogues and that conductivity increases with helix length. They related this enhancement to the increasing helix macrodipole and dielectric constant, which facilitate ion transport. Accordingly, the annealing-induced α-to-β transformation in PLL may suppress this favorable helical electrostatic environment, leading to lower Br^–^ mobility and, consequently, reduced dc conductivity.

**Figure 7:**
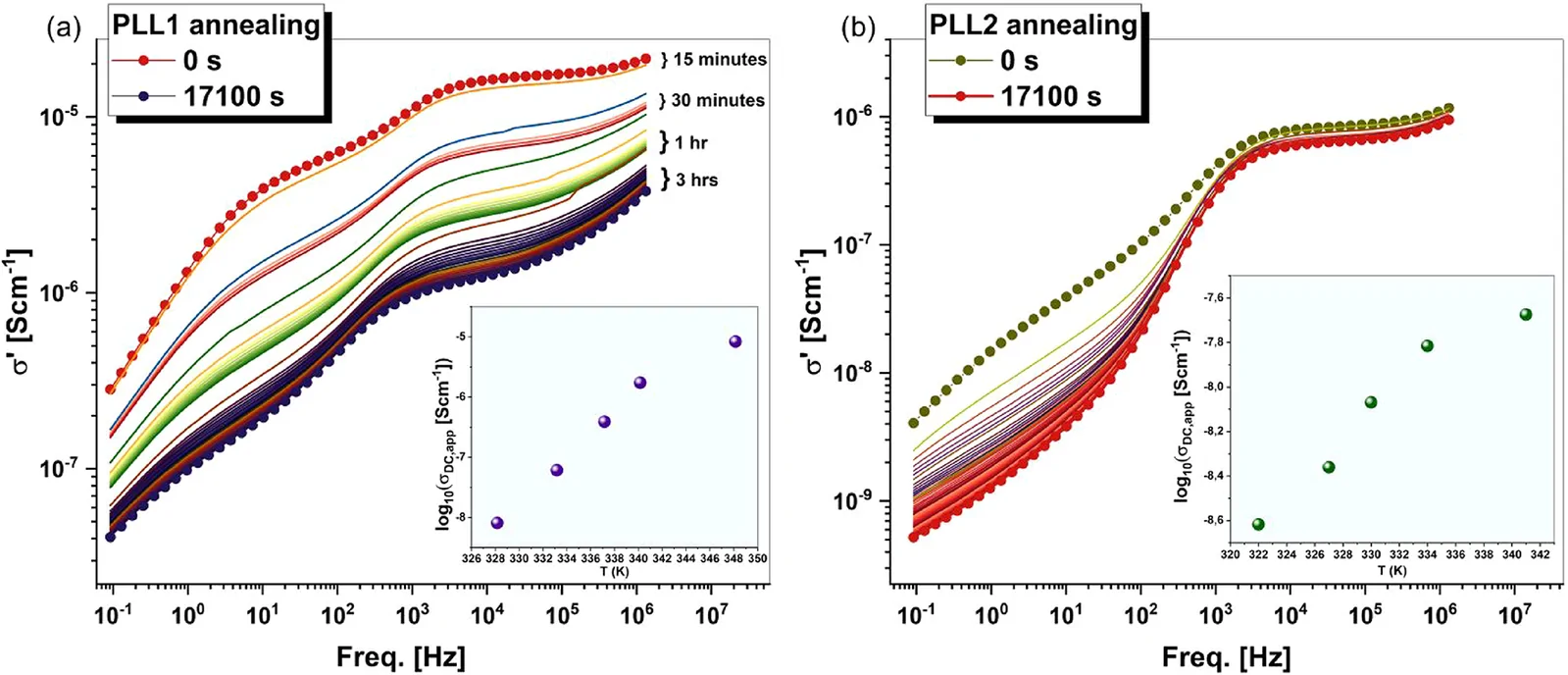
Evolution of the frequency-dependent real conductivity spectra, σ′(f), for the investigated samples during annealing at 403 K for up to 17 100 s: (a) **PLL1** and (b) **PLL2**. The inset in each panel presents log_10_(σ_DC,app_), estimated from the low-frequency plateau/shoulder region of σ′(f), as a function of the corresponding calorimetric *T*_g_ obtained at different annealing stages.

Although the chemical structure of the present α-poly-l-lysine hydrobromide differs from those reported in that work, both systems can be viewed within the broader class of ionic polypeptide-based solid-state electrolytes, in which mobile counterions are transported within a secondary-structure-dependent polymer matrix. Therefore, our results support the view that α-helical organization can be more favorable for DC charge transport than a β-sheet-rich arrangement. Consequently, the conductivity decrease observed here can be rationalized as a result of the thermally induced reorganization from an α-helical electrolyte matrix toward a β-sheet-rich state; the former is associated with a favorable electrostatic environment for Br^–^ migration.

As a final point in our investigations, we plotted log_10_(σ_DC,app_) at the temperatures corresponding to the calorimetrically determined *T*_g_. As shown in the insets of Figure 7a,b, prolonged annealing leads to a simultaneous decrease in *T*_g_ and σ_DC,app_ measured at *T*_g_, with both parameters gradually approaching limiting values. The decrease in σ_DC,app_ at the calorimetric *T*_g_ offers further evidence that charge transport in α-poly-l-lysine hydrobromide is not governed solely by segmental mobility, supporting the interpretation that the α-helical state provides a more favorable electrostatic environment and more continuous pathways for Br^–^ migration than the annealed β-sheet-rich arrangement.

## Conclusions

This study demonstrates that solid-state α-poly-l-lysine hydrobromide (α-PLL) is not a conformationally static system, but undergoes a pronounced thermally induced structural reorganization that directly affects its macromolecular dynamics and charge transport. The freeze-dried PLL samples initially contain mixed populations of α-helical, β-sheet, and β-turn/antiparallel β-sheet conformations, with their relative fractions depending on molecular weight and sample history. Upon heating above the temperature range associated with residual-water removal, both α-PLL systems progressively transform toward β-sheet-rich architectures, although the accompanying supramolecular reorganization is sample dependent. This process is not reversible under the applied experimental conditions and involves not only changes in the secondary structure, but also a reorganization of the H-bonding network and the packing of polypeptide chains. The conformational transformation is thermally activated, with an apparent activation energy of approximately 64 kJ mol^–1^, indicating that β-sheet formation in solid α-PLL requires cooperative rearrangement of H-bonded polypeptide chains. The structural evolution is accompanied by molecular-weight-dependent changes in the two-dimensional hexagonal organization of α-helical chains.

Most importantly, the thermally induced reorganization has a direct impact on the physical properties of α-PLL. Annealing leads to a systematic decrease in *T*_g_ and apparent dc conductivity, σ_DC,app,_ in both samples, demonstrating that segmental dynamics and charge transport are coupled to the conformational state of the polypeptide. The decrease in *T*_g_ is particularly significant, as it shows that the formation of more β-sheet-rich structures does not necessarily rigidify the material. Instead, the rearrangement of H-bonding constraints can reduce the effective constraint density governing cooperative segmental motion. The reduction in apparent DC conductivity provides an additional important insight. Since the dielectric measurements were performed after dehydration of loosely bound water, the suppression of conductivity is unlikely to arise simply from water removal during annealing. Rather, it reflects the effect of conformational reorganization on charge transport in an ionic polypeptide solid electrolyte containing Br^–^ counterions. Although β-sheet formation is accompanied by stronger N–H···O=C H-bonding, these interactions are likely more static and interchain in character, stabilizing β-sheet-rich domains rather than providing efficient long-range transport pathways. In contrast, the α-helical state appears to better support charge transport, possibly through a more favorable electrostatic environment for Br^–^ migration. This conclusion is consistent with recent studies on polypeptide-based solid electrolytes, which showed that α-helical secondary structure can enhance ionic conductivity relative to less ordered or random-coil analogues. Therefore, our results support the view that α-helical organization can promote charge transport in ionic polypeptide-based solid-state materials.

Overall, these results show that the glass transition and dielectric response of solid-state α-poly-l-lysine hydrobromide cannot be regarded as independent of secondary structure. Rather, they arise from the interplay between conformational composition, β-sheet formation, H-bonding topology, counterion mobility, hydration history, and sample-dependent liquid-crystalline-like organization. The physical property relationships identified in this work provide useful guidelines for the design of thermally processed polypeptide-based materials, including bioelectronic and sensing systems. By showing that the solid-state properties of α-PLL are highly sensitive to secondary-structure and supramolecular reorganization, this study provides insight into how these structural features can be used to tune molecular mobility and ion transport in polypeptide-based systems.

## Supplementary Material



## Notes

^1^H NMR spectra of the monomer, Z-protected PLL, and deprotected PLL; FT-IR spectra of **PLL1** and **PLL2** before and after annealing; FT-IR band assignments in the amide I and related spectral regions; comparison of FT-IR spectra of **PLL1** and **PLL2** over the full spectral range at selected temperatures; time-dependent FT-IR spectra used to monitor β-sheet formation; and SEC eluograms of **PLL1** and **PLL2** (PDF)
