# Supplementary material for: Conformation-Determined Segmental Dynamics in α‑Poly‑l‑Lysine Hydrobromide: The Impact of Thermally Induced β‑Sheet Formation on the Glass Transition Temperature
Source: J Phys Chem B. 2026 Aug 6;130(33):8535–49. doi: 10.1021/acs.jpcb.6c03934 (PMC13501882; doi:10.1021/acs.jpcb.6c03934)
Supplement: Supplementary file 1 [file jp6c03934_si_001.pdf]

Conformation-Determined Segmental Dynamics in α-Poly-L-lysine Hydrobromide: The Impact of Thermally Induced β-Sheet Formation on the Glass Transition Temperature

Rafał Bielas[a], Anjana Krishna Sudhakaran Nair Valsala Kumari[b], Krzysztof Witkowicz[c], Anna Mielańczyk[d], Magdalena Tarnacka[b], Anna Janowska[b], Karolina Jurkiewicz[b], Kamil Kamiński[b], Barbara Hachuła[c*], Ewa Kamińska[a*]

[a] *Department of Pharmacognosy and Phytochemistry, Faculty of Pharmaceutical Sciences in Sosnowiec, Medical University of Silesia in Katowice, Jagiellońska 4, 41-200 Sosnowiec, POLAND*

[b] *A. Chełkowski Institute of Physics, Faculty of Science and Technology, University of Silesia in Katowice, 75 Pułku Piechoty 1, 41-500 Chorzów, POLAND*

[c] *Institute of Chemistry, Faculty of Science and Technology, University of Silesia in Katowice, Szkolna 9, 40-006 Katowice, POLAND*

[d] *Department of Chemistry, Silesian University of Technology, B. Krzywoustego 4, 44-100 Gliwice, POLAND*

emails: barbara.hachula@us.edu.pl; ekaminska@sum.edu.pl

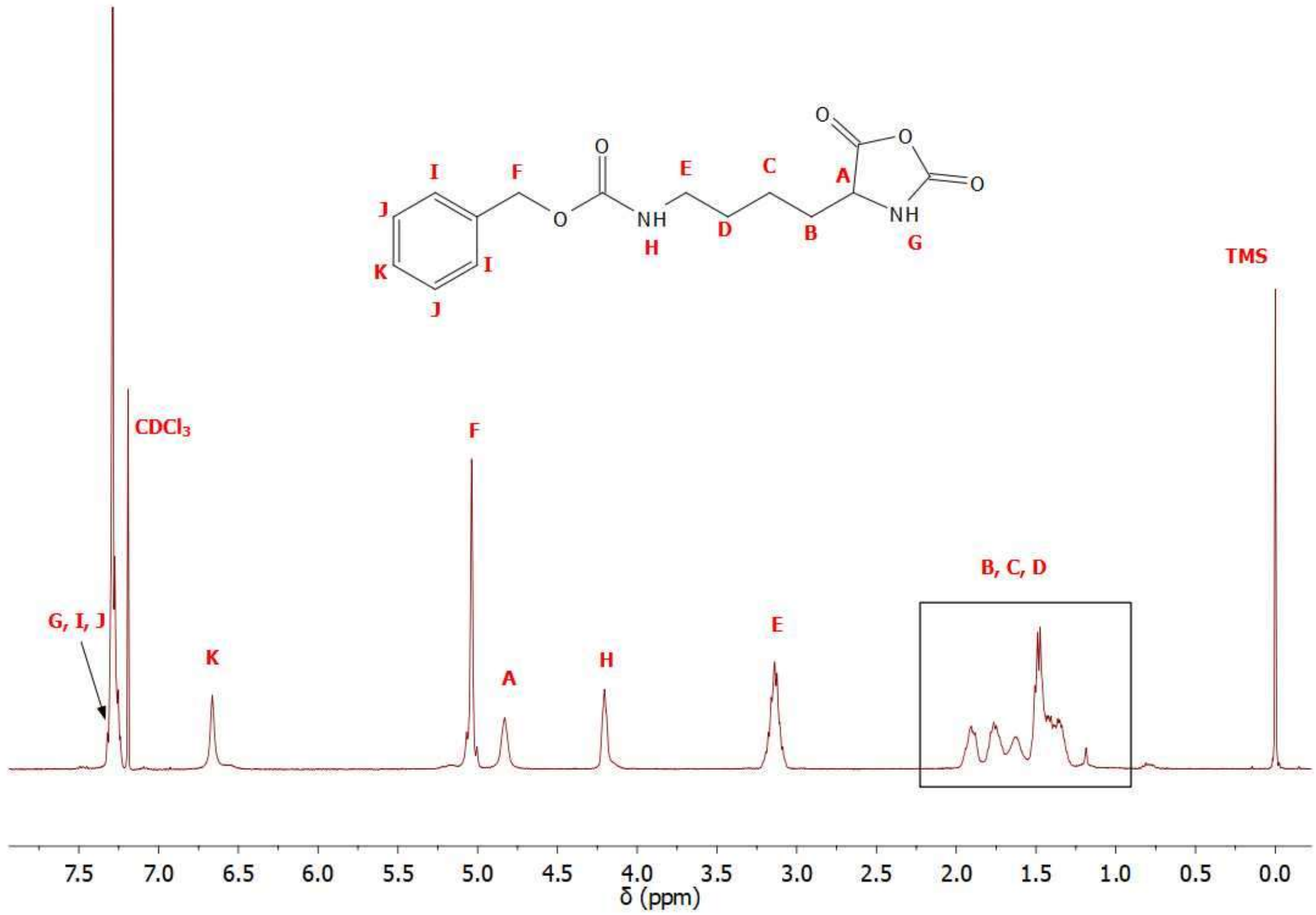

Figure S1. ^{1}H NMR spectrum of Z-Lys NCA.

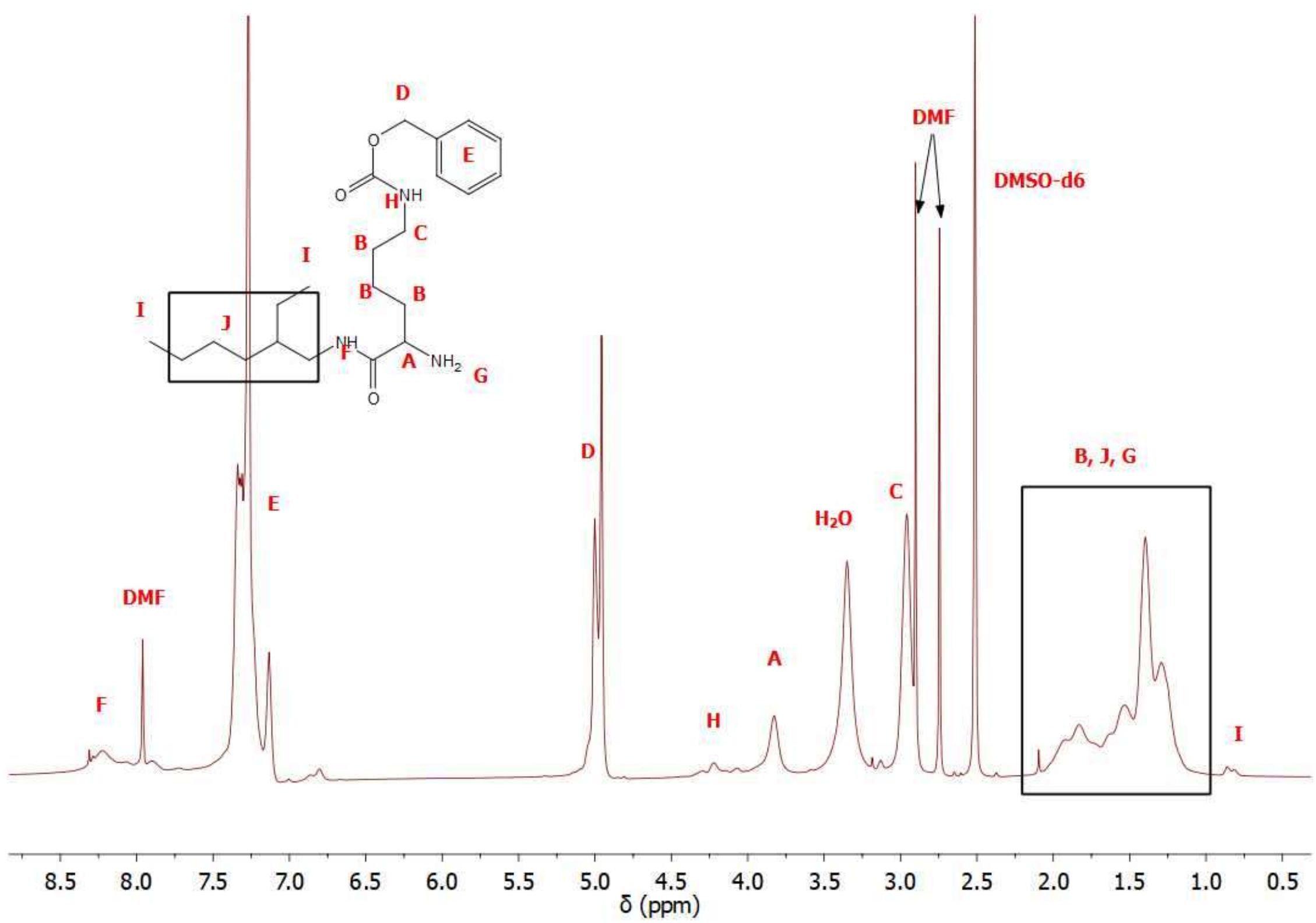

Figure S2. ^{1}H NMR spectrum of Z-protected PLL.

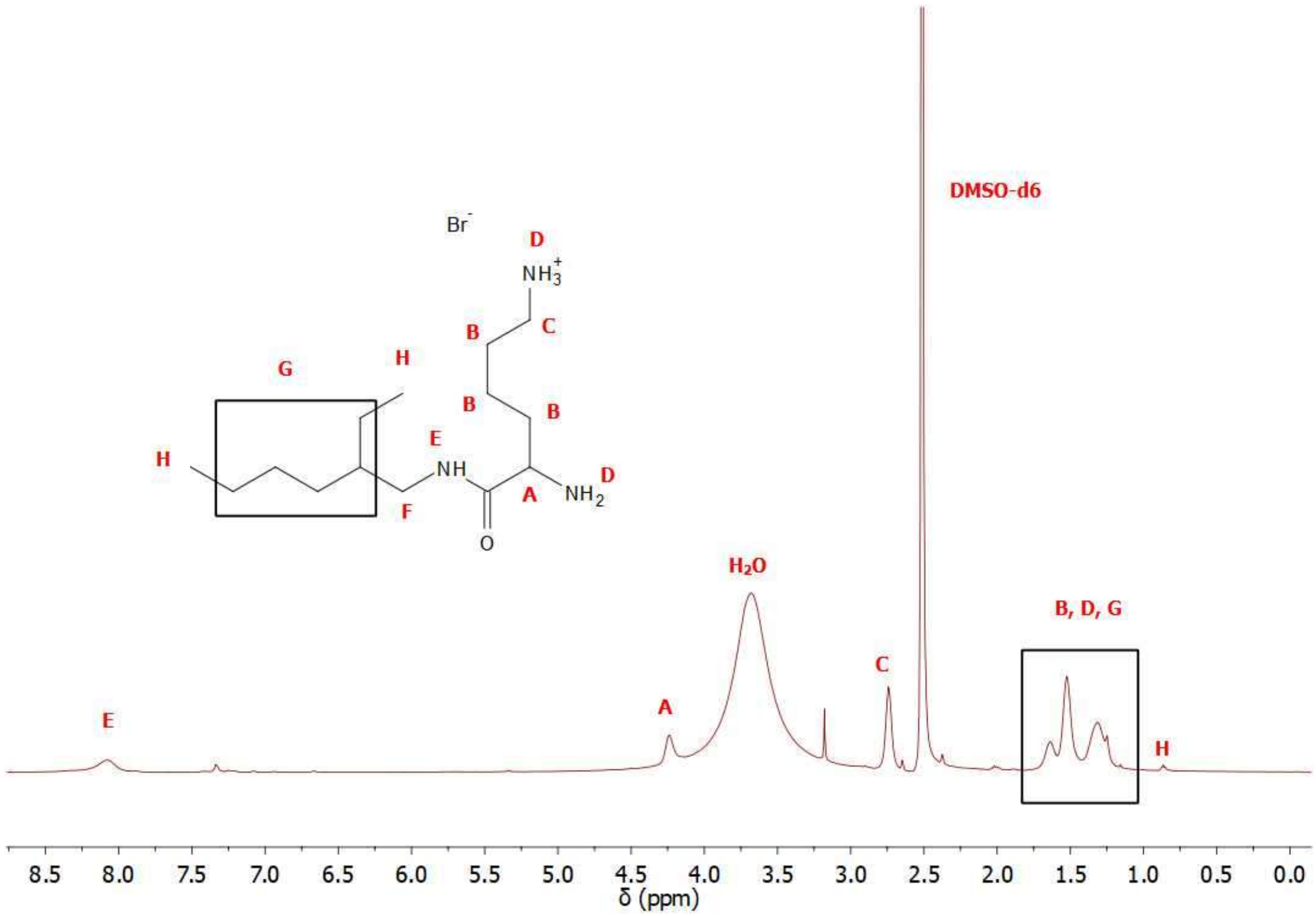

Figure S3. ^{1}H NMR spectrum of deprotected PLL.

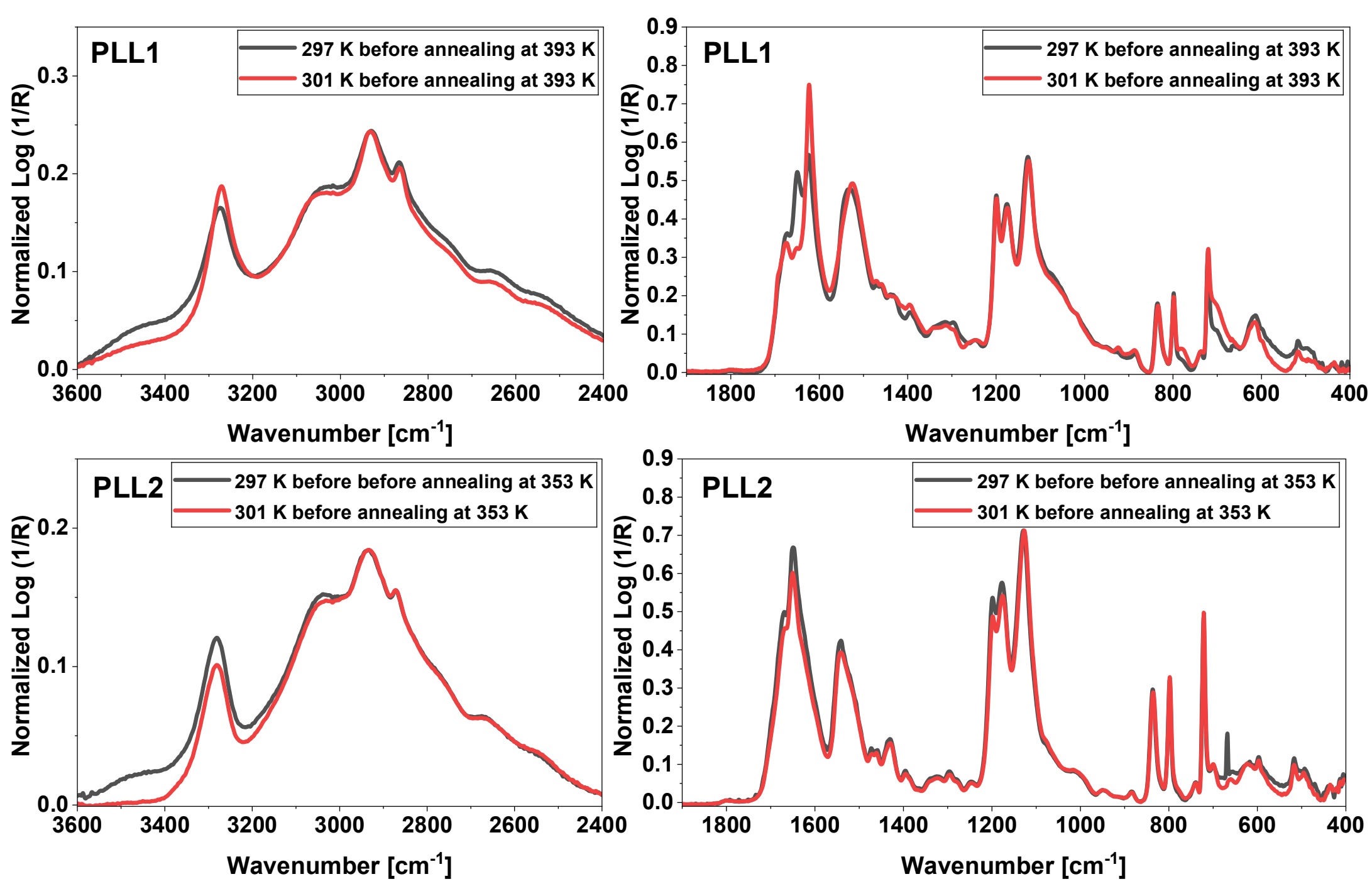

Figure S4. FT-IR spectra of **PLL1** (upper) and **PLL2** (bottom) measured at 297 K before annealing (black) and at 301 K after annealing (red) in the wavenumber ranges: (left) 3600-2400 and (right) 1900-400 cm^{-1}.

As shown in **Figure S4**, heating both **PLL1** and **PLL2** primarily leads to dehydration and changes in the hydrogen-bonding environment, but the spectral response differs between the two samples. In the high-wavenumber region, the broad O–H stretching contribution between 3600 and 3400 cm^{-1} decreases after heating, confirming the removal of loosely bound water. At the same time, the N–H stretching band near 3280 cm^{-1} becomes more clearly resolved, especially in **PLL1**, where its relative intensity increases rather than decreases. This indicates that after water removal, the N–H groups are less masked by the broad water band and participate in a more defined hydrogen-bonding network. In **PLL2**, the same effect is visible but less pronounced, suggesting a less strongly reorganized N–H environment at this temperature.

In the amide region, heating **PLL1** causes a clear redistribution of the amide I components. The α-helical contribution around 1650 cm^{-1} practically disappears after annealing, whereas the lower-wavenumber β-sheet-related component becomes dominant and better resolved. This indicates that dehydration at 393 K is accompanied by substantial structural reorganization of **PLL1** toward a β-sheet-rich arrangement. In **PLL2**, the changes in the amide I region are weaker; the α-helical contribution remains clearly visible, although some redistribution of intensity toward β-sheet-like components may begin. The amide II region around 1540 cm^{-1} also changes after heating, reflecting modifications of peptide-backbone hydrogen bonding and hydration rather than only a loss of water. Overall, annealing of PLL samples removes loosely bound water and reorganizes hydrogen bonds, with a much stronger conformational response in **PLL1** than in **PLL2**.

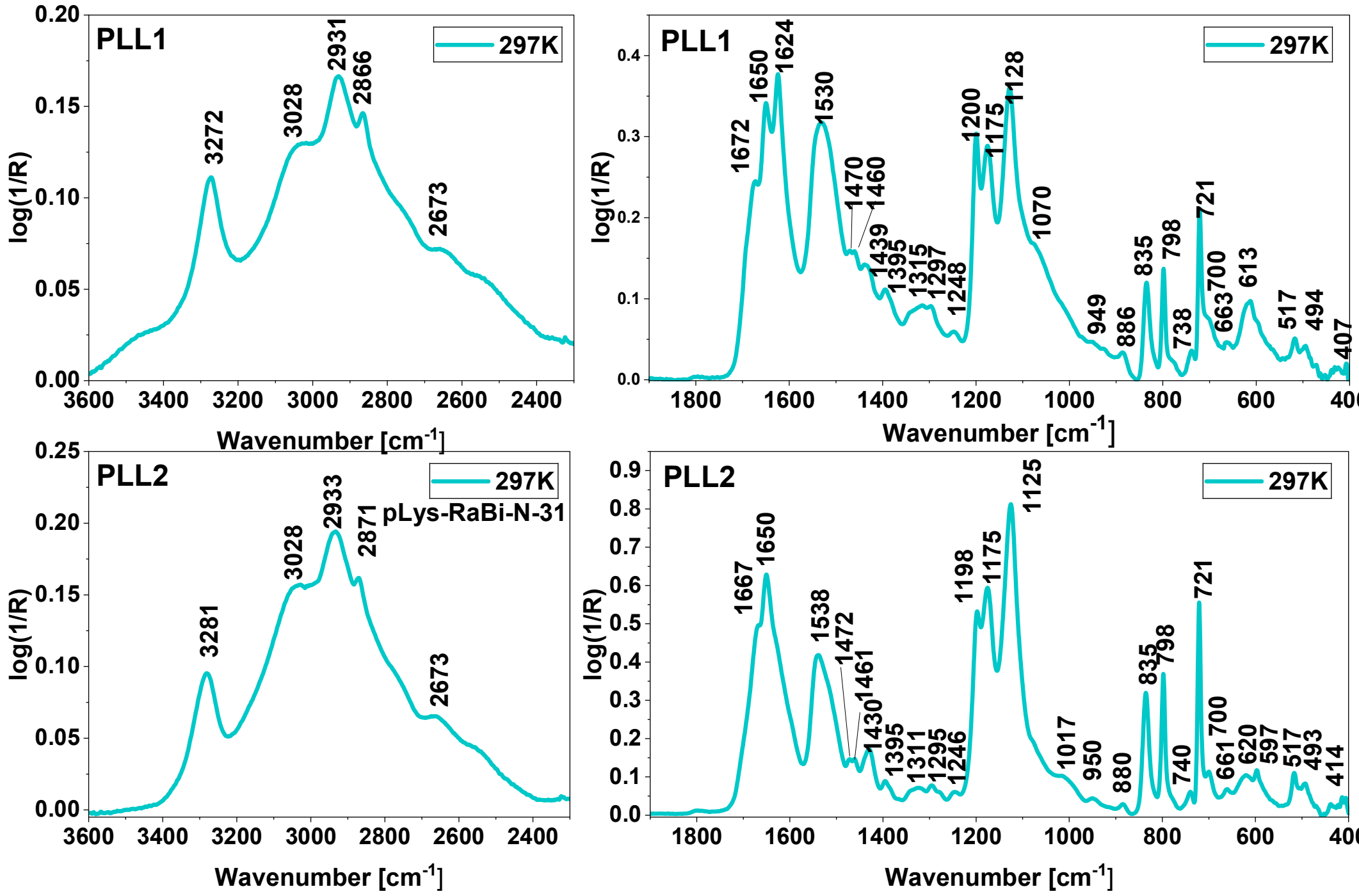

Figure S5. FT-IR spectra of **PLL1** (upper) and **PLL2** (bottom) measured at 297 K in the wavenumber range: (left) 3600-2400 and (right) 1900-400 cm^{-1}.

The FTIR spectrum of the **PLL1** displays several characteristic absorption bands corresponding to the vibrational modes of the polypeptide backbone and the lysine side chains (**Figure S5**). In the high-wavenumber region, the prominent and broad band at 3272 cm^{-1} is assigned to the amide A (N-H stretching) vibration, indicating a well-developed H-bonding network within the polymer. This is followed by the aliphatic region, where the peaks at 2931 cm^{-1} and 2866 cm^{-1} correspond to the asymmetric and symmetric C-H stretching modes of the methylene groups in the lysine side chains. In the amide I region, the spectrum is characterized by a high-frequency signal at 1672 cm^{-1}, associated with β-turns, and a significant peak at 1650 cm^{-1} typical of α-helical conformation.[1,2,3] However, the most intense feature in this region is the band at 1624 cm^{-1}, which provides definitive evidence for a dominant intermolecular β-sheet architecture already present at room temperature. The amide II band at 1530 cm^{-1}, resulting from a combination of the N-H in-plane bending and C-N stretching, further supports the presence of an organized secondary structure.[1,2,3] In the fingerprint region, the weak peaks between 1480 cm^{-1} and 1439 cm^{-1} are attributed to the CH_2 bending and deformation modes of the side chains, while the signals between 1395 cm^{-1} and 1315 cm^{-1} represent C-H wagging and amide III components. The sharp and intense vibrations observed at 1200 cm^{-1}, 1175 cm^{-1}, and 1128 cm^{-1} are characteristic of C-N stretching and skeletal modes, which are highly sensitive to the protonation state of the ε-amino groups.[1] Finally, the lower frequency range from 900 cm^{-1} down to 500 cm^{-1} contains a series of bands related to

N-H out-of-plane bending, CH_2 rocking, and amide IV–VI deformations, all of which reflect the overall long-range structural order of the polylysine sample prior to thermal treatment.

The recorded FTIR spectrum of **PLL2** displays characteristic amide bands of the polypeptide's secondary structure (**Figure S5**). The broad band observed in the 3500–2400 cm^{-1} range, with a maximum at 3281 cm^{-1}, corresponds to the amide A band; this is attributed to N–H stretching vibrations and confirms the involvement of amide groups in extensive H-bonding. The peaks at 2933 cm^{-1} and 2871 cm^{-1} correspond to the asymmetric and symmetric C-H stretching vibrations of the aliphatic methylene groups in the lysine side chains. The most important diagnostic feature is the amide I band located at 1650 cm^{-1}, which arises from the C=O and the C-N stretching and strongly suggests an α-helical conformation.[1,2,3] This is further confirmed by the amide II band at 1538 cm^{-1}, which results from N-H in-plane bending coupled with C-N stretching, a feature typical of these structures. In the mid-range, the signals occurring between 1472 cm^{-1} and 1430 cm^{-1} are attributed to the scissoring vibrations of the CH2 groups, while the amide III region near 1246 cm^{-1} is consistent with a disordered structural arrangement.[1] The region below 1200 cm^{-1} displays intense peaks at 1198 cm^{-1}, 1175 cm^{-1}, and 1125 cm^{-1}, which are due to the C-C skeletal vibrations and the NH_3^+ groups of the side chains. Finally, the lack of a distinct peak around 1630 cm^{-1} confirms that the sample is not in a β-sheet conformation, but exists in a predominantly α-helical state.

References:

(1) Misra, N. K.; Kapoor, D.; Tandon, P.; Gupta, V. D. Vibrational Dynamics and Heat Capacity of Poly(L-lysine). *Polym. J.* **1997**, *29* (11), 914–922. https://doi.org/10.1295/polymj.29.914.

(2) Dzwolak, W.; Smirnovas, V. A Conformational α-Helix to β-Sheet Transition Accompanies Racemic Self-Assembly of Polylysine: An FT-IR Spectroscopic Study. *Biophys. Chem.* **2005**, *115*, 49–54. https://doi.org/10.1016/j.bpc.2005.01.003.

(3) Dzwolak, W.; Muraki, T.; Kato, M.; Taniguchi, Y. Chain-Length Dependence of α-Helix to β-Sheet Transition in Polylysine: Model of Protein Aggregation Studied by Temperature-Tuned FTIR Spectroscopy. *Biopolymers* **2004**, *73* (4), 463–469. https://doi.org/10.1002/bip.10582.

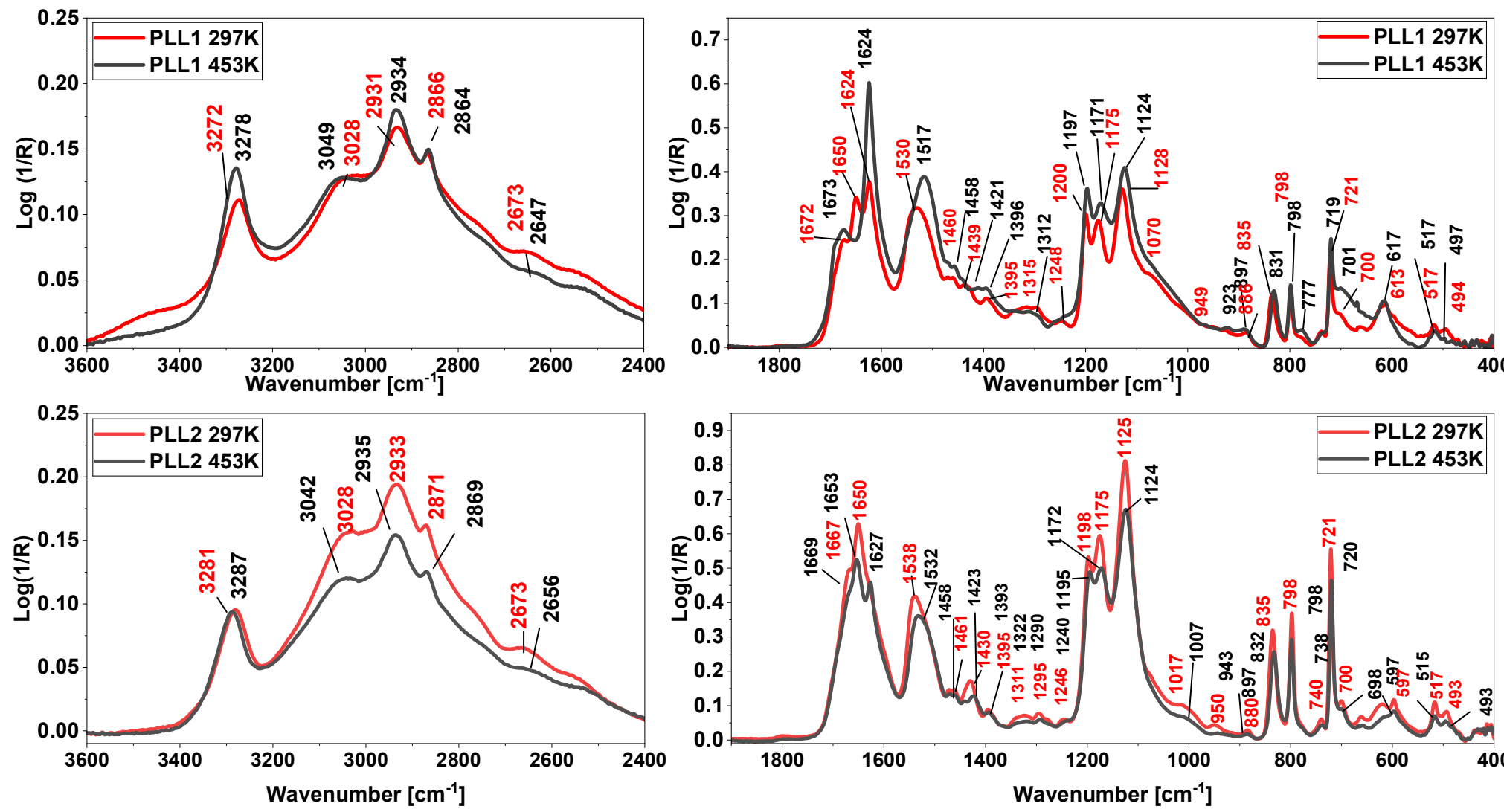

Figure S6. The comparison FT-IR spectra of **PLL1** (upper) and **PLL2** (bottom) measured at 297 K (red) and 453 K (black) in the wavenumber range: (left) 3600-2400 and (right) 1900-400 cm^{-1}.

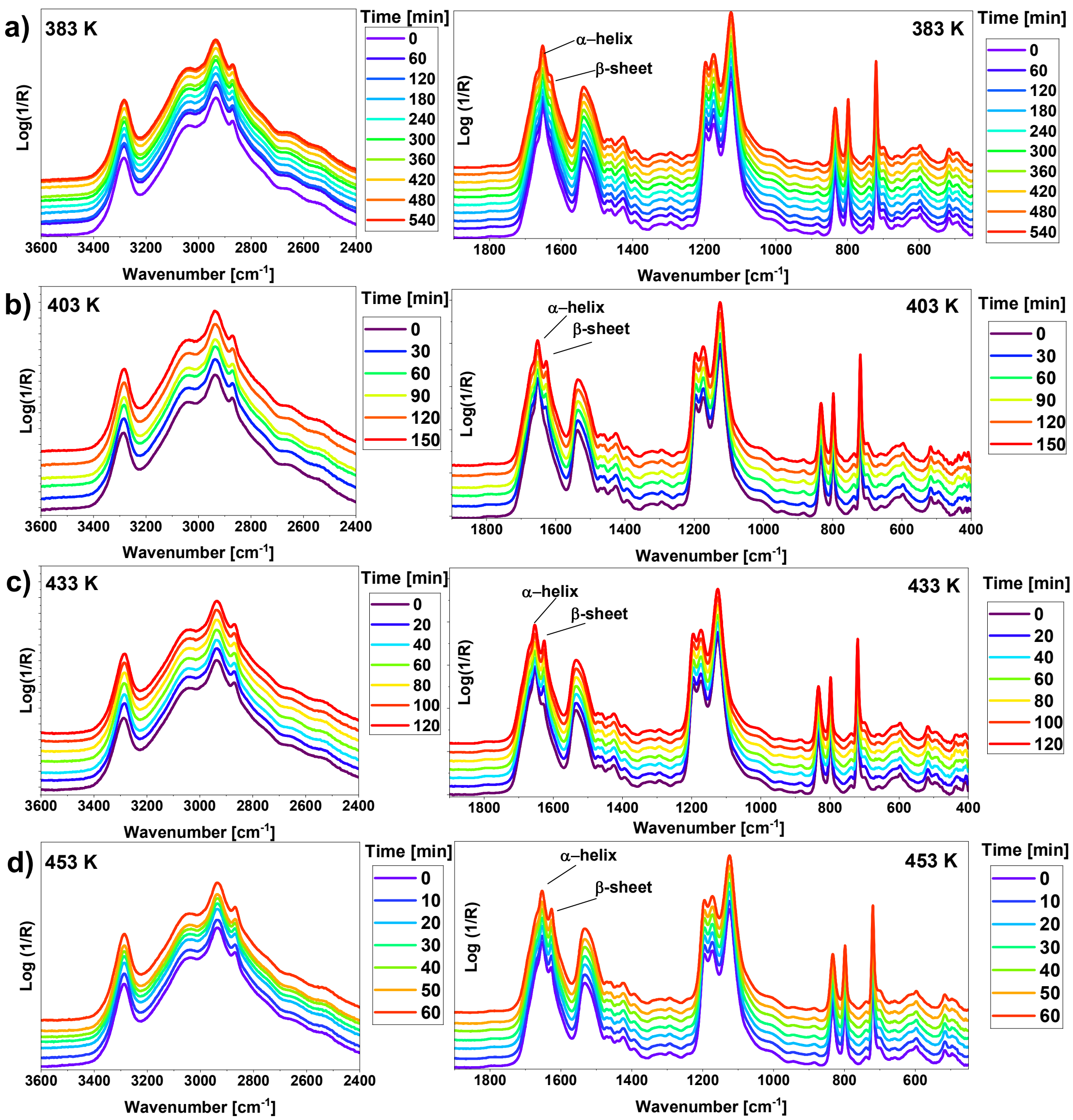

Figure S7. Time-dependent FT-IR spectra of **PLL2** measured at (a) 383 K, (b) 403 K, (c) 433 K, and (d) 453 K in the wavenumber range: (left) 3600-2400 and (right) 1900-400 cm^{-1}.

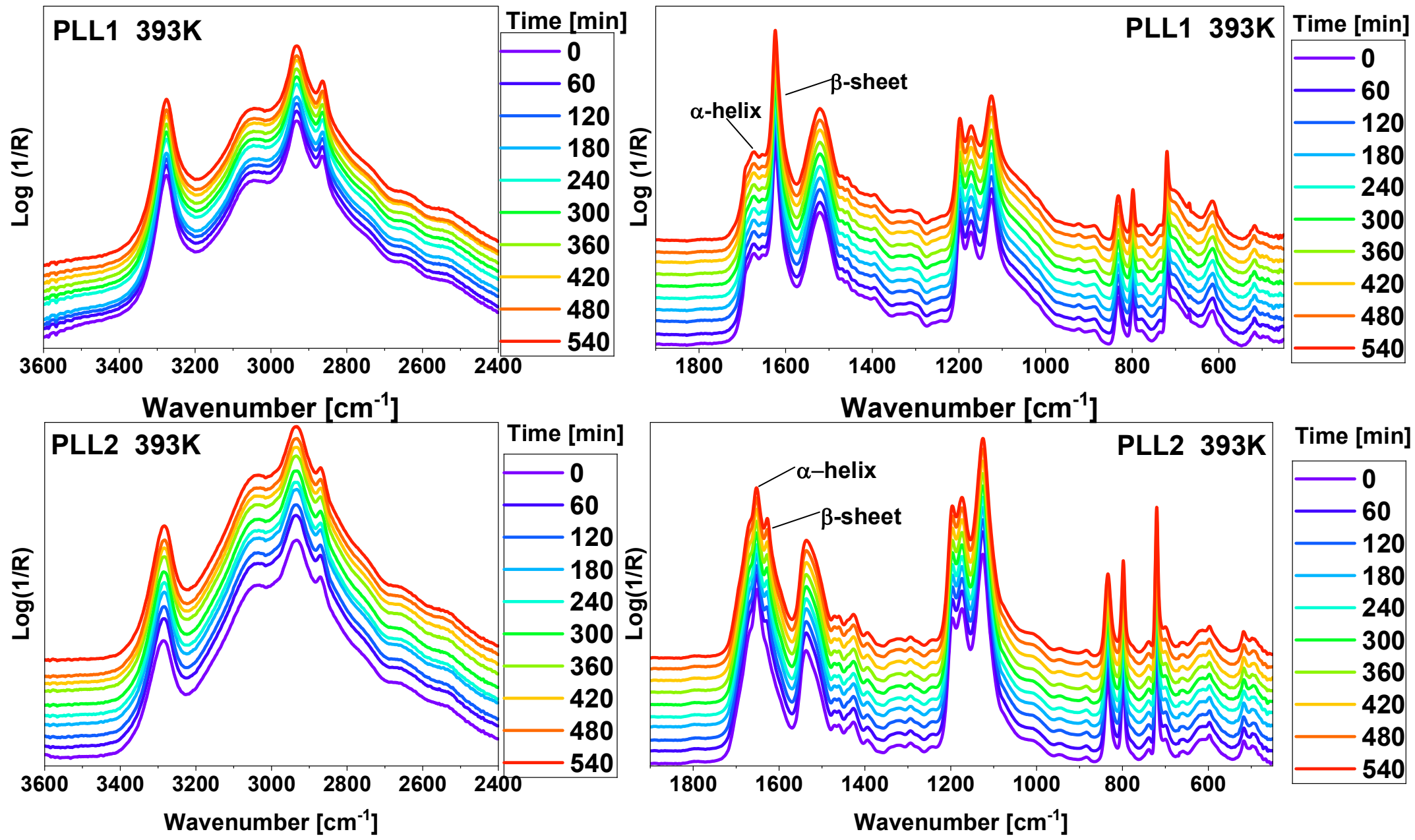

Figure S8. Time-dependent FT-IR spectra of (upper) **PLL1** and (bottom) **PLL2** measured at 393 K in the wavenumber range: (left) 3600-2400 and (right) 1900-400 cm^{-1}.

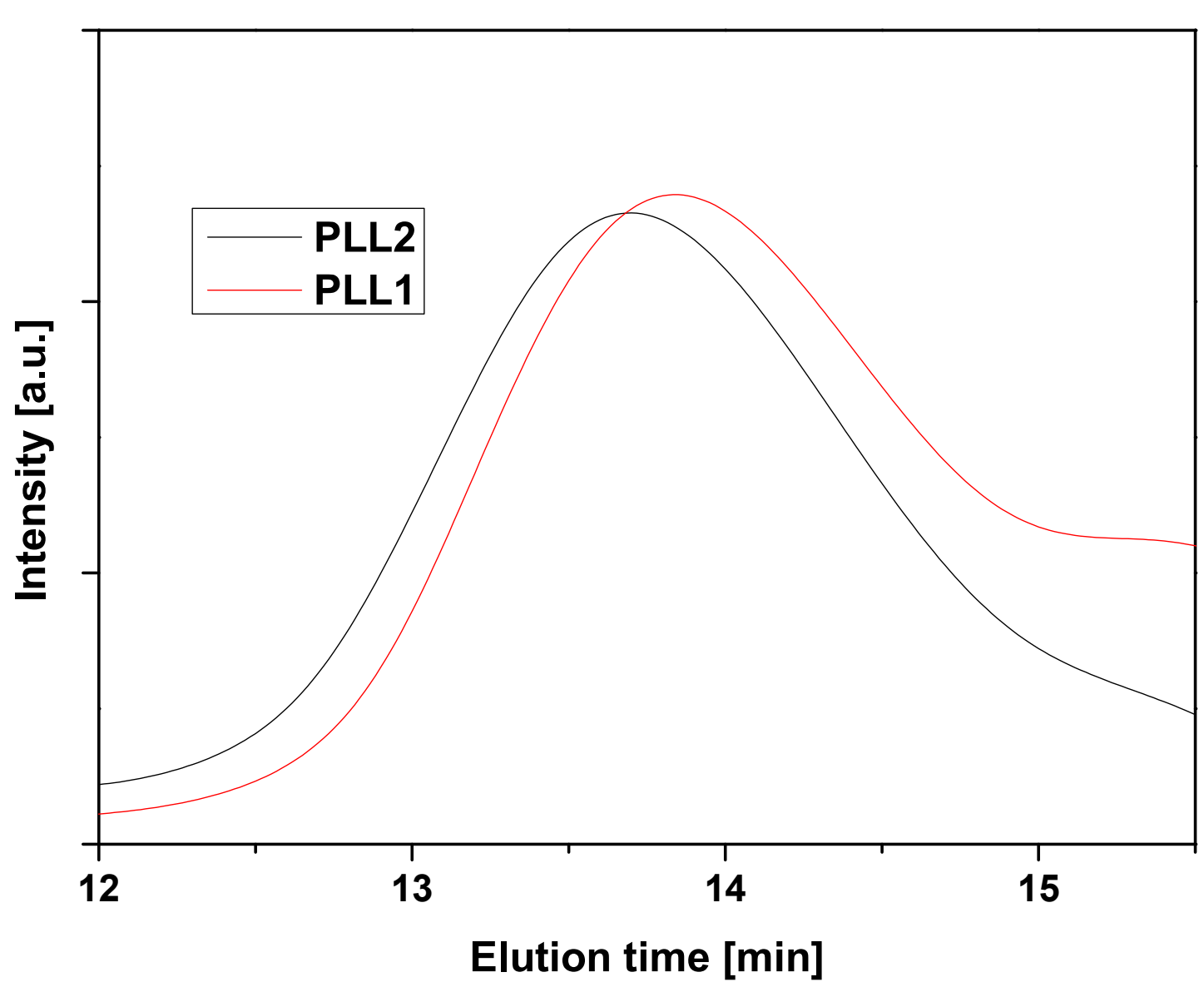

Figure S9. SEC eluograms of **PLL1** and **PLL2**.
